# Peptidomic and transcriptomic profiling of four distinct spider venoms

**DOI:** 10.1371/journal.pone.0172966

**Published:** 2017-03-17

**Authors:** Vera Oldrati, Dominique Koua, Pierre-Marie Allard, Nicolas Hulo, Miriam Arrell, Wolfgang Nentwig, Frédérique Lisacek, Jean-Luc Wolfender, Lucia Kuhn-Nentwig, Reto Stöcklin

**Affiliations:** 1 Atheris SA, Chemin d’Alcire 1, Plan-les-Ouates, Geneva, Switzerland; 2 School of Pharmaceutical Sciences, EPGL, University of Geneva, University of Lausanne, 1, Rue Michel-Servet, Geneva 4, Switzerland; 3 University of Geneva, CMU, 1, Rue Michel Servet, Geneva 4, Switzerland; 4 Atheris Laboratories, Chemin d’Alcire 1, Plan-les-Ouates, Geneva, Switzerland; 5 University of Bern, Institute of Ecology and Evolution, 6, Baltzerstrasse, Bern, Switzerland; 6 SIB Swiss Institute of Bioinformatics, CUI, 7, Route de Drize, Geneva, Switzerland; Universidad de Costa Rica, COSTA RICA

## Abstract

Venom based research is exploited to find novel candidates for the development of innovative pharmacological tools, drug candidates and new ingredients for cosmetic and agrochemical industries. Moreover, venomics, as a well-established approach in systems biology, helps to elucidate the genetic mechanisms of the production of such a great molecular biodiversity. Today the advances made in the proteomics, transcriptomics and bioinformatics fields, favor venomics, allowing the in depth study of complex matrices and the elucidation even of minor compounds present in minute biological samples. The present study illustrates a rapid and efficient method developed for the elucidation of venom composition based on NextGen mRNA sequencing of venom glands and LC-MS/MS venom proteome profiling. The analysis of the comprehensive data obtained was focused on cysteine rich peptide toxins from four spider species originating from phylogenetically distant families for comparison purposes. The studied species were *Heteropoda davidbowie* (Sparassidae), *Poecilotheria formosa* (Theraphosidae), *Viridasius fasciatus* (Viridasiidae) and *Latrodectus mactans* (Theridiidae). This led to a high resolution profiling of 284 characterized cysteine rich peptides, 111 of which belong to the Inhibitor Cysteine Knot (ICK) structural motif. The analysis of *H*. *davidbowie* venom revealed a high richness in term of venom diversity: 95 peptide sequences were identified; out of these, 32 peptides presented the ICK structural motif and could be classified in six distinct families. The profiling of *P*. *formosa* venom highlighted the presence of 126 peptide sequences, with 52 ICK toxins belonging to three structural distinct families. *V*. *fasciatus* venom was shown to contain 49 peptide sequences, out of which 22 presented the ICK structural motif and were attributed to five families. The venom of *L*. *mactans*, until now studied for its large neurotoxins (Latrotoxins), revealed the presence of 14 cysteine rich peptides, out of which five were ICK toxins belonging to the CSTX superfamily. This in depth profiling of distinct ICK peptide families identified across the four spider species highlighted the high conservation of these neurotoxins among spider families.

## 1 Introduction

Venoms consist of small organic molecules, peptides and proteins, optimized for defensive and predatory purpose by Nature through 300 million years of evolution. With 45'828 spider species described to date, [1] each with a venom made of dozens to hundreds of distinct biomolecules, from polyamines to peptides either linear or cysteine-rich, as well as high molecular weight proteins, today these arthropods toxins represent a huge library of potential drug candidates. However toxins from spider venom reported in the UniProt/SwissProt database, are to date limited to 1’446, clearly representing only the tip of the iceberg of this unexplored biodiversity (http://www.uniprot.org/uniprot/) [1–3]. This lack of identified toxins reflects two major drawbacks in spider venom-based studies: the small size of the majority of spider species, the limited amount of venom produced and the difficult sample collection on the one hand; and the lack of optimized methodologies for in depth studies of such complex matrices, on the other hand [4]. This latter aspect is now partially solved with the rise of “-omics” approaches, such as proteomics, transcriptomics and genomics, as well as advances in bioinformatics.

The integrative application of such technologies for the study of venoms initiated the so-called venomics approach [5–7]. Venomics exploits improvements in LC-MS/MS technology, often in conjunction with NextGen mRNA and DNA sequencing, in order to provide a global high resolution profiling of venom and venom gland of the species of interest. This allows considerable amounts of data to be generated, without any discrimination for minor peptides. This approach, combined to dedicated bioinformatics and databases, supports the identification and characterization of an increasing number of toxins.

Addressing still open questions such as the mechanism of recruitment of toxins, or the genetic basis for their hypervariability, venomics helps to better understand and exploit the venomous function [6]. The question of evolution of toxins and venom composition is not new to scientists involved in spider venomics. Today, a commonly accepted answer implies the recruitment of an ancestral gene, followed by mutations generated during duplication and adaptive evolution. However, comparative studies in that sense remain rare [2].

The present study aims to compare the toxin composition of one mygalomorph and three araneomorph spiders (S1 Fig): *Heteropoda davidbowie* (Sparassidae), *Poecilotheria formosa* (Theraphosidae), *Viridasius fasciatus* (Viridasiidae) and *Latrodectus mactans* (Theridiidae). Using a combination of NextGen mRNA sequencing of venom glands (RNA-Seq) coupled to LC-MS/MS venom proteome profiling, a comprehensive dataset of expressed and putative toxins was obtained. In the context of this article however, only the in depth sequence analysis of cysteine-rich toxins expression across distantly related and poorly studied spider species (with the exception of *Latrodectus mactans*) is discussed. A particular emphasis on the disulfide-rich Inhibitor Cysteine Knot (ICK) domain toxins allows a rational comparison of these unrelated species. ICK peptides are known to be ubiquitously recruited by a wide number of diverse species, from plants and microorganisms to animals, and particularly in spider venoms, playing an essential insecticidal role due to their promiscuous action on ion channels [8]. Generally characterized by three disulfide bridges folded following the pattern C_1_-C_4_, C_2_-C_5_, C_3_-C_6_, the two first disulfides form a loop, crossed by the third disulfide bond, forming a knot and thus inferring an unusually high stability. The ICK domain is extraordinarily well conserved across the different spider species studied to date, despite a low similarity of sequences [8–10]. The in depth sequence analysis of ICK toxins expression across distantly related spider species presented here represents an original approach to help elucidating the mechanism of evolution and recruitment of neurotoxins in spiders.

The first selected species for this study is *Heteropoda davidbowie* (Sparassidae), only described in 2008 [11] and distributed in Malaysia, Singapore and Sumatra. The Sparassidae family is poorly studied; only seven toxins from the *Heteropoda venatoria* venom are described in the UniProt/SwissProt database. All these sequences belong to the Huwentoxin-1 family and contain the ICK domain. *Poecilotheria formosa* is a large tarantula (Theraphosidae) of Indian origin. Theraphosidae is one of the most investigated spider families, with 509 entries in the UniProt/SwissProt database, mainly neurotoxins, from 27 species. However, the *Poecilotheria* genus is to date unstudied. *Viridasius fasciatus* is a Viridasiidae spider endemic of Madagascar. No entries for *Viridasius* venom toxins are present in the UniProt/SwissProt database. *Latrodectus mactans* is commonly known as the black widow spider (Theridiidae family). Despite its medical importance for humans, only 19 toxins from the Latrodectus venom have been identified and investigated thus far. Of these, 10 are large proteins (latrotoxins, length > 1000 amino acids), representing the major fraction of Theridiidae venom. These toxins are extremely neurotoxic to insects and mammals due to their activity at the presynaptic level, which leads to massive neurotransmitter release. Nine other so-called Alpha-latrotoxin-associated low molecular weight proteins are described, but their function remains to be determined. To date no neurotoxins containing the ICK domain have been identified in this species, while eight ICK lycotoxins were found in *Latrodectus tredecimguttatus* venom glands transcriptome (UniProt) [1,12].

## 2 Material and methods

### 2.1 Venom collection and venom gland sampling

Spiders and their venoms were purchased from Alphabiotoxine Laboratory sprl (Belgium) (*Heteropoda davidbowie*, *Poecilotheria formosa* and *Latrodectus mactans)*. Venom of *Viridasius fasciatus* was from an own spider stock (L. Kuhn-Nentwig).

Venom was collected by electrical stimulation in glass capillaries. All samples obtained for each species were pooled, frozen at -30°C, freeze-dried in a SpeedVac Plus concentrator (Thermo Savant, Osterville, MA, USA) and stored at -80°C. Venom glands from the same specimen were dissected. *Poecilotheria formosa*, *Viridasius fasciatus* and *Latrodectus mactans* specimen were dissected 0.5, 2, 3 and 4 days after milking (1/4 of specimen dissected at each time-lapse). *Heteropoda davidbowie* specimen were dissected 0.5, 3 and 7 days after milking (3 specimen dissected at each time-lapse) [13,14]. All samples obtained for each species were pooled and stored in RNAlater (Ambion, Thermo Fisher Scientific Inc., Waltham, MA, USA) at -80°C until RNA extraction.

### 2.2 Venom glands transcriptomics

#### 2.2.1 Venom glands mRNA extraction

Venom glands (*Heteropoda davidbowie*: pool of 9 adult specimen; *Poecilotheria formosa*: pool of 10 adult specimen; *Viridasius fasciatus*: pool of 10 adult specimen; *Latrodectus mactans*: pool of 39 adult specimen) were homogenized for RNA extraction, using a Tissuelyser LT (Qiagen, Hilden, Germany) with TRIzol Reagent (Invitrogen, Carlsbad, CA, USA) following the well-established method of Chomcynski and Sacchi [15]. Messenger RNA was purified using Dynabeads mRNA Purification Kit (Invitrogen, Carlsbad, CA, USA), according to manufacturer’s recommendations. Final elution was performed in 20 μl of nuclease free water. The quality of mRNA was assessed using an Agilent 2100 Bioanalyzer (Agilent Technologies, Santa Clara, CA, USA) with Agilent RNA 6000 Pico Kits and the extraction yield was assessed using a Qubit 2.0 fluorometer (Invitrogen, Carlsbad, CA, USA). The mRNA was stored at -80° C prior to subsequent use.

#### 2.2.2 Transcriptome sequencing

cDNA libraries were constructed following the Ion Total RNA-Seq Kit for whole transcriptome protocol (PN 4467091 Rev. C (10/2011)) from Ion Torrent (Life Technologies, Carlsbad, CA, USA). Yield and quality of the libraries were assessed using an Agilent 2100 Bioanalyzer with an Agilent High Sensitivity DNA Kit (Agilent Technologies, Santa Clara, CA, USA) and cDNA were stored at -20°C. The templates were prepared following the Ion OneTouch 200 Template Kit v.2.0 protocol (PN 4478371 Rev. A (06/2012)), and stored at 4°C. Sequencing runs were performed on the Personal Genome Machine following the Ion PGM 200 Sequencing Kit protocol (PN 4474246 Rev. D (06/2012)), using Ion 316 Chip Kit, provided by Life Technologies.

#### 2.2.3 Data assembly

The raw data obtained from the Torrent Suite Software (version 3.6) was converted to FASTQ format and sequencing quality was assessed by aligning the reads to housekeeping genes of arthropods. Sequences belonging to ribosomal RNA (rRNA) were removed by matching reads against public rRNA sequences (Silva database and NCBI BLAST version 2.2.26). Identical reads were removed with CD-HIT [16]. The remaining reads were *de novo* assembled using Mira assembler (version 4) with default parameters for Ion Torrent data [17]. Carry-over contamination between runs in NGS data sequencing is a well-known problem [18,19]. It is magnified for *de novo* assembly of venomous glands in which very few genes are expressed. To eliminate reads coming from a previously sequenced organism, new reads were aligned against previous runs and identical reads were removed.

### 2.3 Venom proteomics

#### 2.3.1 Venom preparation

The crude venom was dissolved (1 mg/ml) in a 2% solvent B solution (90% acetonitrile (Fisher Scientific, Thermo Fisher Scientific Inc., Waltham, MA, USA) + 0.1% trifluoroacetic acid (Pierce-Thermo Scientific Inc., Waltham, MA, USA) in water) in solvent A (0.1% trifluoroacetic acid in water). Sample was extracted on a Sep-Pak Plus C_18_ cartridge (360 mg, 55-105μm, Waters), equilibrated with a solution of 2% solvent B in A. Elution was performed with a solution of 70% solvent B in A. The eluate was collected, frozen at -30°C, freeze-dried and stored at -80°C. The extract was reduced with tris(2-carboxyethyl)phosphine (TCEP) prior to MS analysis: the sample was dissolved in water (1mg/ml) and 1.5μl of TCEP solution (250mM, NaOH was added to recover a pH=4) was added to 15μl of sample. Mixture was heated at 50°C on a heater block (Eppendorf, Hamburg, Germany) for 10 minutes. On cooling, 15μl of water were added and injection was directly performed.

#### 2.3.2 Venom LC-MS/MS analysis

Chromatographic conditions were optimized following the protocol previously established by Eugster et al. (2012) [20]. 5μl of reduced sample were injected into a Thermo Dionex Ultimate 3000 Rapid Separation Quaternary system UHPLC (Thermo Fisher Scientific), equipped with an Acquity 150*2.1, 1.7μm C_18_ reversed-phase column (Waters Corp., Milford, MA, USA) with temperature set at 60°C. A linear gradient from 2 to 45% solvent D was performed over 110 minutes at a flow rate of 0.3 ml/min, where solvent C was 0.1% formic acid in water and D was 0.1% formic acid in acetonitrile.

Detection was performed with a Q-Exactive Plus Orbitrap mass spectrometer (Thermo Fisher Scientific), in positive ionization mode, from *m/z* 200 to 2000. Diisooctyl phthalate C_24_H_38_O_4_ [M+H]^+^ ion (*m/z* 391.28429) was used as internal lock mass. The mass analyzer was calibrated using a mixture of caffeine, methionine-arginine-phenylalanine-alanine-acetate (MRFA), sodium dodecyl sulfate, sodium taurocholate and Ultramark 1621 in an acetonitrile/methanol/water solution containing 1% acid by direct injection. MS/MS analyses were performed on the 5 most intense ions recorded at each scan (Top5 experiment), using stepped normalized collision energy (NCE) auto-switching mode (20; 30; 40). The MS/MS isolation window width was 1 *m/z* Acquisition was performed in centroid mode. Singly-charged ions and ions with unassigned charge state were excluded. Intensity threshold was fixed at 10e^4^ and dynamic exclusion was applied with a window of 30.0 seconds.

In order to match transcriptome sequences against MS/MS protein fragmentation data, all contigs were translated using the Getorf tool on the Galaxyp platform. To retrieve the relevant mass spectra, the X!Tandem module was then used to match MS/MS data against the transcriptomes on the Galaxyp platform (usegalaxyp.org [21,22]). A cutoff of 1 for the e-value was set and all results were manually verified and validated. Only cysteine rich peptides containing a minimum of 2 cys were retained. Manual validation consisted in discarding sequences containing stop codons and signal/noise ratio evaluation.

### 2.4 Data analysis

Putative toxin sequences were retrieved from contigs of the assembled transcriptome using in house built Hidden Markov Model (HMM) profiles for spider toxins. All sequences of Arachnoserver [23] were downloaded and clustered using SiLiX [24]. For each cluster a multiple sequence alignment (MSA) was constructed with MAFFT [25]. For each MSA, the pattern of cysteines was then manually inspected. MSAs that grouped peptides with the same pattern were kept. Those that have heterogeneous patterns were further split until each MSA contains peptides with the same numbers of cysteine with the same spacing. Each MSA was then used to build an HMM profile using hmmer (3.0) [26]. This gave rise to a library of 143 profiles. The profile library was used through Genewise [27] to directly scan the contigs, bypassing the translation step. All the identified contigs were submitted to a selective step to remove those that diverged from canonical sequences due to sequencing errors or partial open reading frames.

Since these results were used for comparative purposes, a strict quality control was applied: contigs represented by less than 10 reads were discarded. Reads per kilobase of contig per million mapped sequence reads (RPKM) values for each retrieved contig were calculated [28] (S2 Table).

All retrieved sequences were submitted to BlastP homology search analysis against the UniProt/SwissProt database [3,29]. An e-value < 1 was set as cutoff.

Alignments were built using Jalview, version 2.9.0b2, using the BLOSUM62 Score algorithm.

## 3 Results

Transcriptome profiling of the four spider species was achieved by whole venom gland mRNA sequencing using a NextGen instrument. Peptidome profiling of the venom from the same animals was achieved by high resolution LC-MS/MS data dependent analysis. All MS/MS spectra were matched against the transcriptome sequences obtained for each species. The transcriptome data were used both for identifying secreted toxin sequences based on HRMS/MS data and to generate a list of putative toxins.

**Fig 1:**
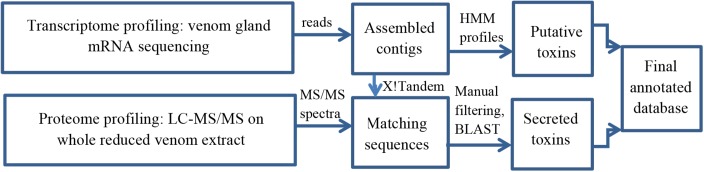
Schematic workflow used to obtain total profiling of venom and venom gland toxins.

Among all sequences identified, only cysteine containing peptides were retained for further comparison in the frame of this study. A schematic workflow of the venom profiling method is depicted in Fig 1.

### 3.1 Venom gland transcriptomics

Raw reads from the venom gland transcriptome sequencing were filtered to remove low quality and ribosomal reads, and the retained sequences were assembled *de novo*. In order to retrieve putative toxin sequences, Hidden Markov Model (HMM) profiles were built for each structural family [30], based on cysteine-rich spider toxins referenced in Arachnoserver, a public domain database [23]. Low confidence sequences, represented by a low number of reads, or containing a frameshift or misassembly, were removed (a minimum of 10 reads was set as cutoff). Each transcriptome generated between 1.7 and 3.9 million individual reads of 120-150 bp mean length, of which only a portion was exploited for assembly into contigs (Table 1), after removing ribosomal RNA and duplicated reads.

**Table 1:** Summary of putative toxins retrieved from the transcriptomic analysis of venom glands from the four spider species.

	*Heteropoda davidbowie*	*Poecilotheria formosa*	*Viridasius fasciatus*	*Latrodectus mactans*
Transcriptome reads	3’629’882	1’711’946	3’766’087	3’602’829
Mean length (bp)	150	138	121	128
Reads used for assembly	993’874	639’643	1’805’619	2’093’250
Contigs	239’749	94’148	330’060	301’423

### 3.2 Venom proteomics

The analysis of *H*. *davidbowie* venom generated a total of 35’806 MS/MS scans. After automatic matching against transcriptome sequences, a total of 1’406 matching spectra were obtained. From these, 824 were found to be good quality spectra based on automatic filtering, 68 of them were retained after manual validation. In this last filtering step only cysteine-rich peptides were retained (> 2 Cys / peptide), sequences containing stop codons were removed and finally quality of the match was assessed by evaluation of the S/N of diagnostic fragments in the MS/MS spectra. Following the same procedure, 103, 9 and 4 sequences were retained for *P*. *formosa*, *V*. *fasciatus* and *L*. *mactans* venoms respectively, leading to a total of 184 validated sequences for the four spiders (Table 2).

**Table 2:** Summary of secreted sequences retrieved from the MS/MS analysis of venom from the four spider species.

	*Heteropoda davidbowie*	*Poecilotheria formosa*	*Viridasius fasciatus*	*Latrodectus mactans*
MS/MS scans	35’806	30’744	30’380	26’793
MS/MS spectra after automatic matching	1’406	1’387	756	486
MS/MS spectra after automatic selection	824	582	65	43
MS/MS sequences manually validated	68	103	9	4

### 3.3 Bioinformatics data analysis

**Fig 2:**
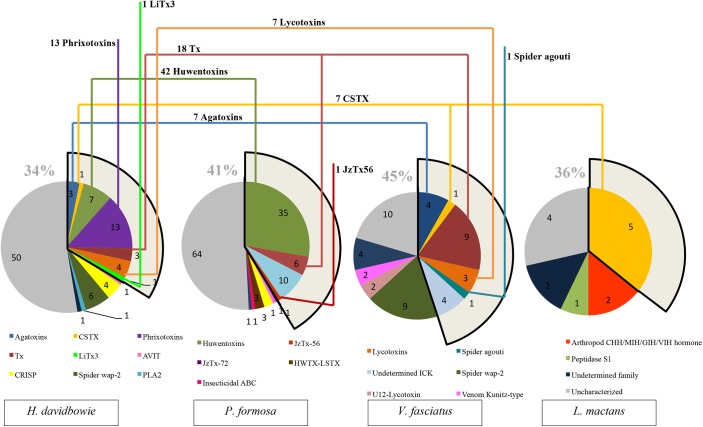
Representation of all peptides retrieved from *H*. *davidbowie* (a), *P*. *formosa* (b), *V*. *fasciatus* (c) and *L*. *mactans* (d) venom and venom glands transcriptome analysis.

**Fig 3:**
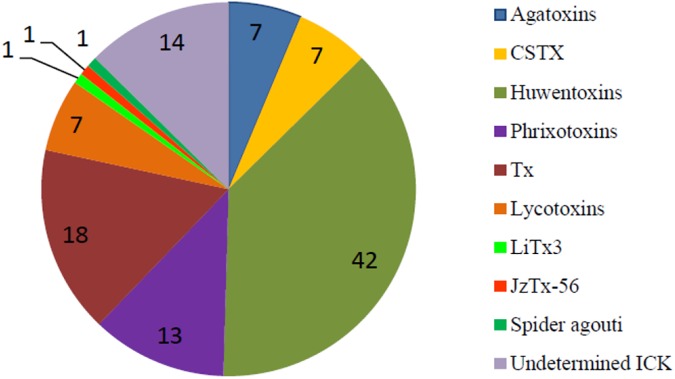
Representation of structural families belonging to the ICK motif discovered in *H*.*davidbowie*, *P*. *formosa*, *V*. *fasciatus*, *L*.*mactans*.

In order to extract putative cysteine-rich toxins from each assembled transcriptome, the HMM models for 143 spider toxins structural families were built and initially used to scan the contigs libraries [31,32]. This led to extract 29, 37, 41 and 10 putative sequences from *H*. *davidbowie*, *P*. *formosa*, *V*. *fasciatus* and *L*. *mactans* transcriptomes respectively and leaded a total of 117 sequences for the four spiders. Secondly, these putative toxins were added to the 183 identified by proteomics in the secreted venoms. Only 17 sequences were shared in both datasets, confirming the presence of these toxins in the venom. Undetected toxins in the venom can be due to a low level of expression, leading to a quantity of protein below the limit of the MS profiling method. Besides, the presence of posttranslational modifications (PTM) or the incorrect prediction of the propeptide cleavage sites, could result in undetected matching. This however cannot be interpreted as an unequivocal absence of the remaining putative toxins in the venom as already reported in other venomics studies [33]. Thirdly, a BLAST homology search was performed on the 284 non-redundant retrieved sequences from the four spider species against the UniProt/SwissProt protein database to determine the structural family to which they belong. 55% of the sequences could be classified by this mean, while 45% remained uncharacterized (S1 to S6 Tables). Families as defined in UniProt/SwissProt were used as reference to classify the new retrieved sequences, based on cysteine pattern and sequence homology between cysteines [32,34]. Among them, finally, a list of 111 secreted and/or putative toxins containing the inhibitor cysteine knot structural motif was retrieved to allow a rational comparison among the species studied (Tables 3 and 4, Fig 2). These results are summarized in Table 3. All the structural families identified in each spider are represented in Fig 3, while the distribution of ICK families for each spider is represented in Fig 2. All the predicted mature sequences presenting the ICK structural motif are listed in S1 Table, named following the guidelines published by King and colleagues [34,35].

**Table 3:** Summary of toxins retrieved from the proteomic and transcriptomic analysis of venom and venom glands from the four spider species.

	*Heteropoda davidbowie*	*Poecilotheria formosa*	*Viridasius fasciatus*	*Latrodectus mactans*	Total
MS/MS sequences manually validated	68	103	9	4	184
Uncharacterized secretory peptides	50	64	8	4	126
Homologous to arthropod toxins	17	39	1	0	57
Putative toxins retrieved by HMM detected in the transcriptome	29	37	41	10	117
Putative toxins retrieved by HMM detected in venom	2	14	1	0	17
Total sequences	95	126	49	14	284
ICK toxins	32	52	22	5	111

**Table 4:** Summary of ICK toxins identified in the four spiders and ICK families repartition.

	*Heteropoda davidbowie*	*Poecilotheria formosa*	*Viridasius fasciatus*	*Latrodectus mactans*
Identified peptides	95	126	49	14
Identified ICK toxins	32	52	22	5
% ICK in the venom	34%	41%	45%	36%
ICK families	7	3	4	1
ICK families new to the considered spider family	6	0	4	1

As shown in Fig 2, the analysis of the *H*. *davidbowie* venom revealed the presence of 11 distinct structural toxin families. Out of these 7 ICK families were identified, comprising 32 toxins, which represent the 34% of all identified peptides for this species. From these, 13 belonged to the Phrixotoxin family, seven were Huwentoxin-like, four were homolog to Lycotoxins, three belonged to the Tx-3 family, while three were homologous to the Agatoxins family. The *P*. *formosa* venom analysis highlighted the presence of eight distinct structural toxin families. Out of the 52 ICK toxins, representing the 41% of all identified peptides for this species, 42 belonged to three families: 35 were Huwentoxin-like, six belong to the Tx-2 family and one was classified in the JzTx-56 family. The venom of *V*. *fasciatus* contains eight distinct toxin families. Out of these 22 ICK toxins (45% of all identified peptides for this species), 17 could be distributed in the Tx-3 (nine toxins), Lycotoxins (three toxins), Omega-agatoxins (four toxins), Spider agouti family (one toxin) and other CSTX superfamily (one toxin). The investigation of the *L*. *mactans* venom allowed to highlight the presence of three families of peptide toxins and to discover five novel ICK toxins belonging to the CSTX superfamily. The presence of ICK toxins, representing 36% of all the cysteine-rich peptides identified in this venom was surprising, as to date only large Latrotoxins are reported in the UniProt/SwissProt database for this species. However, ICK toxins were previously highlighted in the venom glands transcriptome of *Latrodectus tredecimguttatus*, where eight lycotoxins were identified [12].

All the identified toxins filtered according to our workflow were thus efficiently classified into various known groups, but they all were found to have new sequences and presented only similarity to known families.

## 4 Discussion

**Fig 4:**
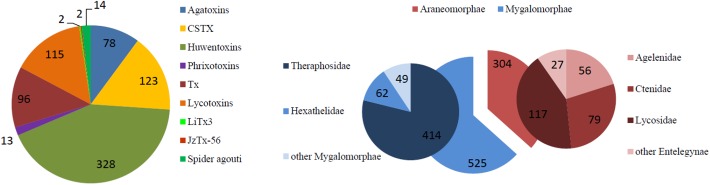
Representation of ICK neurotoxins from spider venoms and venom glands reported in the SwissProt/UniProt database by spider families. On the left side an illustration of all the reported ICK toxins by family. On the right side a representation of the number of ICK toxins reported for each spider family.

From all the 284 retrieved toxins, 111 were identified as belonging to the ICK domain families. These were selected and analyzed in order to evaluate and compare the recruitment of this class by the four spiders. The ICK domain is a characteristic triple disulfide bridge architecture, which results in an enhanced stability of peptides when subjected to extreme conditions, such as high temperature, low pH, or exposure to proteases [9]. ICK peptides are ubiquitously recruited by all species, from plants to microorganisms, and particularly by venomous animals such as cone snails, scorpions, hymenoptera, as well as spiders. However, this class of peptides includes a multitude of distinct families, each typically considered as peculiar to a defined species. Sequence similarity is generally low, but the cysteine pattern is well conserved across all the phyla [36]. ICK toxins in spider venoms are responsible for multiple neurotoxic activities, typically insecticidal, relating to defensive and predatory purposes. These toxins have been studied for their interaction with potassium, calcium and sodium ion channels, Transient Receptors Potential (TRP) channels and ryanodine receptors, as well as for their antifungal action [10,37–39]. To date, 829 ICK toxins from spiders are reported in the UniProt/SwissProt database. As illustrated in Fig 4 (right side), 50% of these were identified in Theraphosidae spiders, reflecting the focus of researchers on large tarantulas or spiders thought to be dangerous for humans. This also demonstrates the lack of global studies on neurotoxin recruitment across all spiders. As a result, as illustrated on the left side, the large majority of reported ICK toxins to date, belong to the Huwentoxins family, originally identified and prevalent in Theraphosidae venoms. Nine distinct structural families containing the ICK motif were identified across the four spiders selected here according their phylogenetic diversity (Fig 3) and are discussed more in detail.

### 4.1 Huwentoxins

A total of 42 new Huwentoxin-like sequences were identified. Of those, 35 originate from the *P*. *formosa* tarantula, showing similarity with different previously described theraphotoxins. Seven additional Huwentoxins originate from *H*. *davidbowie* and showed similarity with previously described sparatoxins from *H*. *venatoria* but also with different theraphotoxins.

The Huwentoxin family is a heterologous family of ICK toxins, widely recruited by venomous spiders, which act on ion channels, mainly potassium and sodium voltage-gated channels, but also activate TRPV capsaicin receptors. This family is represented by 328 toxins reported in UniProt. Mainly found in theraphosid venoms, Huwentoxins have also been identified in *Macrothele gigas* venom (Hexathelidae), *Trittame loki* transcriptome (Barychelidae), *Heteropoda venatoria* (Sparassidae) transcriptome and in the venom of three *Phoneutria sp*. (Ctenidae) [3,40–48]. Multiple alignment obtained for these sequences, highlighting similarity level, is illustrated at S2 Fig.

### 4.2 Lycotoxins

This analysis allowed the identification of seven Lycotoxins: four were identified in *H*. *davidbowie* and three in *V*. *fasciatus* venom glands transcriptomes. First isolated from the venom of *Geolycosa* sp. (Lycosidae), Lycotoxins were further identified in the transcriptome of *Lycosa singoriensis* (Lycosidae) and electrophysiological studies revealed an ion channel inhibitor activity [3,49–51]. A total of 172 Lycotoxins are to date reported in UniProt. Multiple alignment obtained for these sequences, highlighting similarity, is illustrated at S3 Fig. The characteristic cysteine pattern is maintained, however the similarity level between the newly identified sequences and described Lycotoxins is low (between 40 and 62.5% identity and e-values ≥ 9.9e-17), suggesting that an activity profiling cannot be attributed to these toxins by similarity.

### 4.3 CSTX superfamily

The CSTX superfamily is made of several toxins, mostly from undetermined families.

**Fig 5:**
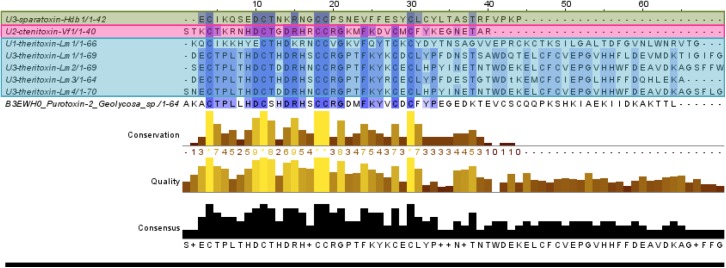
Alignment of the sequences homologous to the CSTX superfamily: five sequences from *Latrodectus mactans*, one from *Viridasius fasciatus* and 1 from *Heteropoda davidbowie* were retrieved. The last reference sequence is the purotoxin-2 from *Geolycosa sp*. (Lycosidae) (Uniprot, accession number B3EWH0)

Isolated from *Cupiennius salei* spider (Ctenidae), CSTX toxins have been extensively studied by Kuhn-Nentwig and colleagues for their ICK structure inferring a calcium channel blocking activity [52]. They are also found in other Entelegynae families such as Miturgidae [53] and Zodariidae [54] Four sequences possessing the ICK domain identified in the *Latrodectus mactans* venom showed similarity with purotoxin-2 from *Geolycosa sp*, while one was similar to U1-ctenitoxin-Cs1a from Cupiennius salei. One sequence identified in *Heteropoda davidbowie* and one in *Viridasius fasciatus* venom matched the latartoxin-1a from *Lachesana tarabaevi* (Zodariidae) and the CSTX-14 toxin from *Cupiennius salei* (Ctenidae) respectively (Fig 5).

A total of 123 toxins belonging to the CSTX superfamily are to date reported in UniProtKB [3,55–58].

### 4.4 Omega and beta/delta-agatoxins

One Omega-agatoxin–like was identified in *H*. *davidbowie* venom, and showed homology with omega-agatoxin-1A, isolated from the *Agelenopsis aperta* (Agelenidae) spider venom. Two other Beta/delta-agatoxins were highlighted, homologous to mu-agatoxin-Aa1f. Sequences identified in *V*. *fasciatus* venom were homologous to the U18-ctenitoxin-Pn1a and the U7-ctenitoxin-Pr1a, from *Phoneutria* spp venoms.

Mainly identified in Ctenidae and Agelenidae venom, but also in the transcriptome of *Lycosa singorinsis* (Lycosidae), 33 Omega-agatoxins are to date well characterized and reported in UniProtKB. These toxins are studied in particular for their calcium channel blocking activity, which leads to a blockage of neuromuscular transmission, causing a rapid paralysis [51,59,60]. Beta/delta-agatoxins were mainly identified in Agelenidae spiders, but also in *Hardonyche infensa* (Hexathelidae), and show an activity on Na_v_ channels which induces paralysis in insects [61]. Multiple alignment obtained for these sequences, highlighting similarity level, is illustrated at S4 and S5 Figs. Sequence U1-sparatoxin-Hdb5 showed a high homology with Omega-agatoxin-1A, suggesting that it probably acts as Ca_v_ channel blocker [62], while U8-ctenitoxin-Vf1 and 2 were found highly similar to U18-ctenitoxin-Pn1a, whose molecular target remains to our knowledge not determined.

### 4.5 Phrixotoxins

The 13 newly identified Phrixotoxins were all discovered in the venom of *H*. *davidbowie*, representing the first Phrixotoxins identified in Araneomorphae spiders. In contrast, it was surprising that no Phrixotoxins were discovered in the *P*. *formosa* tarantula venom since all the 13 Phrixotoxins reported to date originate from spiders of the same family (Theraphosidae). Targeting mainly sodium and potassium voltage-gated channels, toxins belonging to this family are being studied for their potential as analgesic drugs [3,44,47,48,63,64]. Multiple alignment obtained for these sequences, highlighting similarity level, is illustrated at S6 Fig. Due to the low homology values however, a prediction of the activity of these newly described Phrixotoxins based on sequence similarity, would be speculative.

### 4.6 Tx 1, 2 and 3 families

The 6 sequences identified in the *P*. *formosa* venom are closely related to the U32-theraphotoxin-Cg1a from a Theraphosidae spider, while the 9 sequences identified in *V*. *fasciatus* venom are homologous to different Ctenitoxins from *Phoneutria* (Ctenidae). The three sequences identified in *H*. *davidbowie* venom are related to three different Ctenitoxins.

Spider Tx families 1 to 3 have thus far been identified from Ctenidae and Theraphosidae venom. 40 toxins from these families, mainly acting on ion channels as inhibitors, are to date described in the UniProt database [3,45,65]. Multiple alignment obtained for these sequences, highlighting similarity level, is illustrated at S7 Fig.

### 4.7 Other ICK peptides

One unique sequence showing a homology with LiTx-3 toxins was detected in both *H*. *davidbowie* venom and venom glands transcriptome. LiTx3 toxin family was firstly described in *Loxosceles intermedia* (Sicaridae), as a main insecticidal compound in venom, predicted to act on Na_v_ channels [66]. The low similarity level between the newly identified U5-sparatoxin-Hdb1 and the previously described U2-sicaritoxin-Li1b (37.3% identity, 2 e-2 e-value) however, suggests that this peptide may not target the same ion channel.

One unique sequence related to JzTx-56 toxins was identified by HMM in the *P*. *formosa* venom glands transcriptome. JzTx-56 toxins were identified in the *Chilobrachys jingzhao* (Theraphosidae) venom glands transcriptome, and remain poorly studied to date [44].

One sequence issued from *V*. *fasciatus* venom glands transcriptome was found homologous to the Spider agouti family, previously identified in *Lycosa singoriensis* spider. The activity profile of these toxins remains however uncharacterized to our knowledge.

A total of 14 toxins possessing the ICK domain were detected but could not be classified into a determined toxin family. The presence of these poorly characterized toxins indicates a lack in spider venom toxins systematics and experimental activity studies.

To summarize, as illustrated in Table 4, the present study allowed to assign six new ICK families to Sparassidae spiders. The *Poecilotheria formosa* venom analysis revealed three ICK families already known in Theraphosidae venom. This is not surprising since Theraphosidae family was extensively studied and the majority of the known ICK neurotoxins were identified in theraphosid venom. Viridasiidae is a new and completely unstudied family. Thus the four ICK families identified in the *Viridasius fasciatus* venom are new to this family. The analysis of *Latrodectus mactans* allowed to assign 5 CSTX neurotoxins, described for the first time in a Theridiidae venom.

## 5 Conclusion and perspectives

This study illustrates a rapid method for the efficient high resolution profiling of venoms and venom glands. The data analysis focused on cysteine-rich toxins from the venom proteome and venom glands transcriptome of four phylogenetically distant spider species: *Heteropoda davidbowie*, *Poecilotheria formosa*, *Viridasius fasciatus* and *Latrodectus mactans*. The *H*. *davidbowie* venom contains 32 ICK peptides, distributed in seven different families, six of them never reported previously in the Sparassidae. *P*. *formosa* venom revealed the highest number of ICK toxins, the large majority (35 out of 52) belonging to the Huwentoxin family. The analysis of *V*. *fasciatus* venom led to the characterization of 22 novel ICK toxins, distributed in 4 families. This is the first study of neurotoxins from the venom of a viridasid spider. The five CSTX toxins identified in the *L*. *mactans* venom, were to our knowledge the first ICK toxins of this family identified in this species.

While this study was conducted on individual samples from four families only, it is to our knowledge, the first comparative analysis on four phylogenetically distant species focusing on ICK toxins. The methodology also generated important information on other classes of toxins, such as linear peptides and proteins, mostly uncharacterized, that opens the door to further investigations. This approach generated valuable data on expressed and putative toxins for in depth and sensitive comparison purposes and seems well adapted to a much larger screen of biodiverse spider venoms across all families. This would provide a better understanding of toxin recruitment among spiders but also extent of intra species variability.

In addition this will also provide novel bioactive peptides, available for pharmacological research and potentially representing new leads candidates.

## Supporting information

S1 Table Summary of ICK toxins retrieved from the transcriptomic and proteomic analysis of venom glands and venom from the four spider species. (DOCX)

S2 Table RPKM values of the ICK toxins retrieved for each species. (DOCX)

S3 Table Characterized sequences from *H*. *davidbowie* venom glands and venom. (DOCX)

S4 Table Characterized sequences from *P*. *formosa* venom glands and venom. (DOCX)

S5 Table Characterized sequences from *V*. *fasciatus* venom glands and venom. (DOCX)

S6 Table Characterized sequences from *L*. *mactans* venom glands and venom. (DOCX)

S7 Table GenBank accession number of the characterized sequences from *H*. *davidbowie*, *P*. *formosa*, *V*. *fasciatus* and *L*. *mactans* venom glands and venom. (DOCX)

S1 Fig Phylogenetic tree of the spider families for which at least one publication on an elucidated venom compound is available. These represent 32 families out of 114. Modified from Kuhn-Nentwig et al. [1] with adaptations following Polotow et al. [2].
(PDF)

S2 Fig Alignment of the sequences homologous to the Huwentoxin family: 7 sequences from *Heteropoda davidbowie* and 35 sequences from *Poecilotheria formosa*. The last reference sequence is the Omega-sparatoxin-Hv1b from *Heteropoda venatoria* (Sparassidae) (Uniprot, accession number P61790).
(PDF)

S3 Fig Alignment of the sequences homologous to the Lycotoxin family: 4 sequences from *Heteropoda davidbowie* and 3 from *Viridasius fasciatus* were retrieved. The last reference sequence is the U6-lycotoxin-Ls1g from *Lycosa singoriensis* (Lycosidae) (Uniprot, accession number B6DCV8).
(PDF)

S4 Fig Alignment of the sequences homologous to the Beta/delta-agatoxin family: 2 sequences from *Heteropoda davidbowie* were retrieved. The last reference sequence is the Mu-agatoxin-Aa1f from *Agelenopsis aperta* (Agelenidae) (Uniprot, accession number P11062).
(PDF)

S5 Fig Alignment of the sequences homologous to the Omega-agatoxin family: 1 sequence from *Heteropoda davidbowie* and 4 from *Viridasius fasciatus* were retrieved. The last reference sequence is the U7-ctenitoxin-Pr1a from *Phoneutria reidyi* (Ctenidae) (Uniprot, accession number P84031).
(PDF)

S6 Fig Alignment of the sequences homologous to the Phrixotoxin family: 13 sequences from *Heteropoda davidbowie* were retrieved. The last reference sequence is the beta/kappa-theraphotoxin-Cg2a from *Chilobrachys guangxiensis* (Theraphosidae) (Uniprot, accession number Q2PAY4).
(PDF)

S7 Fig Alignment of the sequences homologous to the spider Tx family: 3 sequences from *Heteropoda davidbowie*, 6 sequences from *Poecilotheria formosa* and 9 from *Viridasius fasciatus* were retrieved. The last reference sequence is the Kappa-ctenitoxin-Pn1a from *Phoneutria nigriventer* (Ctenidae) (Uniprot, accession number O76200).
(PDF)
