# Supplementary material for: Peptidomic and transcriptomic profiling of four distinct spider venoms
Source: PLoS One. 2017 Mar 17;12(3):e0172966. doi: 10.1371/journal.pone.0172966 (PMC5357004; doi:10.1371/journal.pone.0172966)
Supplement: S1 Table — (DOCX) [file pone.0172966.s001.docx]

| Proposed Name | Predicted/ Detected Mature Sequence | Retrieved by HMM | Detected by MS/MS | Length | Cys number | Match found in Uniprot | Specie | Family | % Identity | e-value |
| --- | --- | --- | --- | --- | --- | --- | --- | --- | --- | --- |
| U1-sparatoxin-Hdb1 | TEAFYMSIEDAALDTVMARDDDKKDCVGHMGWCAWTDSECC |  | x | 41 | 4 | mu-agatoxin-Aa1f | *Agelenopsis aperta* | beta/delta agatoxin | 52.4 | 7.70E-01 |
| U1-sparatoxin-Hdb2 | MGWCAWTDSECCEGYRCSSPYPAAAAELPSGGLRGK |  | x | 36 | 4 | mu-agatoxin-Aa1f | *Agelenopsis aperta* | beta/delta agatoxin | 47.4 | 2.10E+00 |
| U1-sparatoxin-Hdb3 | AKSLPEGAPCDGDKDDCQCYGKWHKCRCPWFWEDGPCRCAWGLKHTCITKLSCPNKGEWGLDWRSEEERSPC | x |  | 72 | 10 | omega-agatoxin-1A | *Agelenopsis aperta* | type I omega-agatoxin | 72.4 | 1.70E-45 |
| U3-sparatoxin-Hdb1 | ECIKQSEDCTNKRNGCCPSNEVFFESYCLCYLTASTRFVPKP | x |  | 42 | 6 | latartoxin-1a | *Lachsana tarabaevi* | CSTX | 48.5 | 4.80E-03 |
| U4-sparatoxin-Hdb2 | ERDCRKFMGLCKSDDDCCPHLMCYKYGWCGWDGS |  | x | 34 | 6 | beta-theraphotoxin-Cm2a | *Ceratogyrus marshalli* | huwentoxin-1 | 68.8 | 1.80E-10 |
| U4-sparatoxin-Hdb6 | DLGDALFSAEDQQNLQERDCRKFMGLCKSDDDCCPHLMCYKYGWCG |  | x | 46 | 6 | beta-theraphotoxin-Cn2a | *Ceratogyrus marshalli* | huwentoxin-1 | 66.7 | 1.40E-09 |
| U4-sparatoxin-Hdb13 | DCKWIFGSCETSDVCCEGWVCSKGLCKYKLWR | x |  | 32 | 6 | omega-sparatoxin-Hv1a | *Heteropoda venatoria* | huwentoxin-1 | 55.6 | 3.40E-07 |
| U4-sparatoxin-Hdb14 | CIGHLDSCLTASCCPGYKCKCDSIDCLCV | x |  | 29 | 8 | U24-theraphotoxin-Cg1a | *Chilobrachys guangxiensis* | huwentoxin-1 | 100 | 2.70E+00 |
| U4-sparatoxin-Hdb15 | GDDKENCKYWFDSCETEGECCDNWTCHNGICKTIVKAVVRSHKTWSKV |  | x | 48 | 6 | omega-sparatoxin-Hv1b | *Heteropoda venatoria* | huwentoxin-1 | 55.6 | 6.50E-04 |
| U4-sparatoxin-Hdb16 | GDDKENCKYWFDSCETEGECCDNWTCHNGICKIKIIL |  | x | 37 | 6 | omega-sparatoxin-Hv1b | *Heteropoda venatoria* | huwentoxin-1 | 55.6 | 3.20E-04 |
| U4-sparatoxin-Hdb17 | MNLHSTMFCLHSCETEGECCDNWTCHNGICKIKIIL |  | x | 36 | 6 | omega-sparatoxin-Hv1b | *Heteropoda venatoria* | huwentoxin-1 | 60 | 5.90E-04 |
| U6-sparatoxin-Hdb11 | DCIGHMGWCAWTDSECCVGYRCKLWCRKCVPLVTRSTTAALCPDSSI | x |  | 47 | 8 | beta/kappa-theraphotoxin-Cg2a | *Chilobrachys guangxiensis* | phrixotoxin | 71.4 | 7.90E-04 |
| U6-sparatoxin-Hdb12 | DCVGHMGWCAWTDSECCEGYRCKLWCRKI | x |  | 29 | 6 | beta/kappa-theraphotoxin-Cg2a | *Chilobrachys guangxiensis* | phrixotoxin | 73.9 | 9.30E-07 |
| U6-sparatoxin-Hdb13 | DCIGWMGWCSGKELKCCEGHVCSLWCKKKLG | x |  | 31 | 6 | beta/kappa-theraphotoxin-Tp2a | *Thrixopelma pruriens* | phrixotoxin | 71.4 | 8.20E-09 |
| U6-sparatoxin-Hdb14 | DCIGWMGWCSGKDKKCCKGNVCNLWCRYKADVLDLISLIR | x |  | 40 | 6 | kappa-theraphotoxin-Cg2b | *Chilobrachys guangxiensis* | phrixotoxin | 54.2 | 6.70E-06 |
| U6-sparatoxin-Hdb15 | DCNGWTAWCNNCCEDFVCNIWCSLKQALKE | x |  | 30 | 6 | beta-theraphotoxin-Gr1a | *Grammostola rosea* | phrixotoxin | 52.4 | 6.00E-03 |
| U6-sparatoxin-Hdb16 | DDDCIGWMGLCSSSEKKCCEGYACEVWCKYD | x |  | 31 | 6 | kappa-theraphotoxin-Cg2b | *Chilobrachys guangxiensis* | phrixotoxin | 68 | 1.50E-08 |
| U6-sparatoxin-Hdb17 | DDDKKDCIGHMGWCAWTDSECCVGYRCKLWCRKIIDWLGD | x |  | 40 | 6 | beta/kappa-theraphotoxin-Cg2a | *Chilobrachys guangxiensis* | phrixotoxin | 73.9 | 4.80E-06 |
| U6-sparatoxin-Hdb18 | DDDCIGWMGLCSSSEKKCCEGYACEVWCKYDLDGEKV | x |  | 37 | 6 | kappa-theraphotoxin-Cg2a | *Chilobrachys guangxiensis* | phrixotoxin | 65.5 | 7.50E-10 |
| U6-sparatoxin-Hdb19 | DDDKKDCVGHMGWCAWTDSECCVGYRCKLWCRKI |  | x | 34 | 6 | beta/kappa-theraphotoxin-Cg2a | *Chilobrachys guangxiensis* | phrixotoxin | 72.7 | 4.60E-06 |
| U6-sparatoxin-Hdb20 | GDDEDCIGWMGWCSGKDKKCCKGNVCNLWCRYKADV |  | x | 36 | 6 | kappa-theraphotoxin-Cg2b | *Chilobrachys guangxiensis* | phrixotoxin | 54.2 | 6.00E-06 |
| U6-sparatoxin-Hdb21 | GDDQTCIGWMGWCSGKNIGCCEGYKCELWCKYA |  | x | 33 | 6 | kappa-theraphotoxin-Cg2a | *Chilobrachys guangxiensis* | phrixotoxin | 63 | 2.30E-07 |
| U6-sparatoxin-Hdb22 | MARDDDKKDCVGHMGWCAWTDSECCVGYRCKLMC |  | x | 34 | 6 | beta/kappa-theraphotoxin-Cg2a | *Chilobrachys guangxiensis* | phrixotoxin | 69.6 | 4.50E-03 |
| U6-sparatoxin-Hdb23 | EDDKDCNGWTAWCNNCCEDFVCNMWCSLKQALKE |  | x | 34 | 6 | beta-theraphotoxin-Gr1a | *Grammostola rosea* | phrixotoxin | 52.4 | 4.10E-03 |
| U5-sparatoxin-Hdb1 | DCVKDGGSCNNGETCCQGGKYKRICGCEAWNPGHCTCHDDCQVC | x | x | 44 | 10 | U2-sicaritoxin-Li1b | *Loxosceles intermedia* | LiTx3 | 37.3 | 2.00E-02 |
| U8-sparatoxin-Hdb1 | GQCADVWKRCGNGVECCCNRSCICNVTRTKCKCHRFHEVSHGYTAECLV | x |  | 49 | 10 | kappa-ctenitoxin-Pn1a | *Phoneutria nigriventer* | Tx3 | 44.4 | 3.30E-04 |
| U8-sparatoxin-Hdb2 | AIEVCGQLYKECSKRFKCCENR | x |  | 22 | 4 | omega-ctenitoxin-Pr1a | *Phoneutria reidyi* | Tx3 | 50 | 9.00E+00 |
| U8-sparatoxin-Hdb3 | AIEVCGQLYKECSKRFKCCENRPCKCNKRGER | x |  | 32 | 6 | omega-ctenitoxin-Pr1a | *Phoneutria reidyi* | Tx3 | 55.6 | 6.60E-05 |
| U9-sparatoxin-Hdb1 | QCIKLEGECTKNKDNCCAEHRCRCYDKYVNGIKTEVR | x |  | 37 | 6 | U6-lycotoxin-Ls1f | *Lycosa singoriensis* | U6-lycotoxin | 51.6 | 5.20E-05 |
| U9-sparatoxin-Hdb2 | QCIKLEGECTKNKDNCCAEHRCRCYDKYVNWNKNR | x |  | 35 | 6 | U6-lycotoxin-Ls1f | *Lycosa singoriensis* | U6-lycotoxin | 62.5 | 3.60E-04 |
| U9-sparatoxin-Hdb3 | QCIKLEGECTKNKDNCCAEHRCRCYDKYVNGIKTEVRCWCFEKDVTYKPTFEIK | x |  | 54 | 8 | U6-lycotoxin-Ls1e | *Lycosa singoriensis* | U6-lycotoxin | 55.3 | 9.90E-17 |
| U9-sparatoxin-Hdb4 | QCTPQEHRCFKGAPKCCGGFDCQCYTPIVNGVKEEPTCWCNEPNVIYEYAFKAQY | x |  | 55 | 8 | U6-lycotoxin-Ls1g | *Lycosa singoriensis* | U6-lycotoxin | 40.4 | 9.80E-08 |
| U1-theraphotoxin-Pf1 | EIQECGHLHEKCNPGPPSTNTCCRGLQCRYGSCLVQV | x | x | 37 | 6 | U27-theraphotoxin-Cg1a | *Chilobrachys guangxiensis* | _ | 71.4 | 5.80E-15 |
| U1-theraphotoxin-Pf2 | CLPAGSACSGPLQKIPCCGTCSRKKCT | x |  | 27 | 6 | omega-theraphotoxin-Hs1a | *Haplopelma schmidti* | _ | 66.7 | 4.20E-08 |
| U1-theraphotoxin-Pf3 | RCLPAGSACSGPIQKIPCCGTCSRKKCT | x | x | 28 | 6 | omega-theraphotoxin-Hs1a | *Haplopelma schmidti* | _ | 66.7 | 7.60E-09 |
| U1-theraphotoxin-Pf4 | GCLKEGKWCPKSAPCCRPLVCKGPSIKQKKCT | x | x | 32 | 6 | U12-theraphotoxin-Hs1a | *Haplopelma schmidti* | _ | 87.1 | 1.10E-19 |
| U1-theraphotoxin-Pf5 | KGCIKEGKWCPKSAPCCRPLVCKGPSIKQKKCT | x |  | 33 | 6 | U12-theraphotoxin-Hs1a | *Haplopelma schmidti* | _ | 87.1 | 9.00E-20 |
| U1-theraphotoxin-Pf6 | GCLKEGKWCPKSAPCCRPLVCKGPSIKQKKCTKP | x |  | 34 | 6 | U12-theraphotoxin-Hs1a | *Haplopelma schmidti* | _ | 87.1 | 1.40E-19 |
| U1-theraphotoxin-Pf7 | CLKEGKWCPKSAPCCRPLVCKGPSNKAEESCTINHLNSKA | x |  | 40 | 6 | U12-theraphotoxin-Hs1a | *Haplopelma schmidti* | _ | 84.6 | 3.70E-12 |
| U2-theraphotoxin-Pf1 | SCIKEWQICKNDCECCGMSTLCKSSWIDG | x |  | 30 | 6 | hainantoxin-XV-5 | *Haplopelma hainanum* | _ | 75.9 | 5.40E-16 |
| U2-theraphotoxin-Pf2 | SCIKEWQICKNDCECCGMSTLCKSSWIDGREIKLCRNEGGKLKKVLHFIQKSVSKIKSCKK | x |  | 61 | 8 | hainantoxin-XV-4 | *Haplopelma hainanum* | _ | 86.9 | 4.30E-42 |
| U2-theraphotoxin-Pf3 | ACSKQIGEKCSGNCDCCGSTVVCGSVYVGGKEEKFCSDKSSNNAALNTAGKGINAVSNMFSFCWG | x |  | 65 | 8 | hainantoxin-XVIII-6 | *Haplopelma hainanum* | _ | 66.7 | 6.30E-29 |
| U4-theraphotoxin-Pf1 | DCRKFMGLCKSDDDCCPHLMCYKYGWCGWDGSV | x | x | 33 | 6 | beta-theraphotoxin-Cm2a | *Ceratogyrus marshalli* | huwentoxin-1 | 66.7 | 1.20E-09 |
| U4-theraphotoxin-Pf2 | GDSENENLQERDCRKFMGLCKSDDDCCPHLMCYKYGCVDGWQC |  | x | 43 | 7 | beta-theraphotoxin-Cm2a | *Ceratogyrus marshalli* | huwentoxin-1 | 56.3 | 5.50E-06 |
| U4-theraphotoxin-Pf3 | ECRWLFGGCTKDADCCKHLGCTRSYPQYCGWDLTV | x | x | 35 | 6 | U21-theraphotoxin-Cg1a 3 | *Chilobrachys guangxiensis* | huwentoxin-1 | 75.7 | 4.60E-19 |
| U4-theraphotoxin-Pf4 | ECRWLFGGCTKDADCCKHLGCRRSYPQYCGWDLTV |  | x | 35 | 6 | U21-theraphotoxin-Cg1a | *Chilobrachys guangxiensis* | huwentoxin-1 | 78.4 | 7.60E-21 |
| U4-theraphotoxin-Pf5 | AECRWMFGSCKEDSDCCKHLGCRRKAPQYCAWDGTV | x | x | 36 | 6 | U2-theraphotoxin-Cg1a | *Chilobrachys guangxiensis* | huwentoxin-1 | 74.3 | 2.40E-18 |
| U4-theraphotoxin-Pf6 | AECRWMFGSCKEDSDCCKHLGCRRDAPQYCAWDGTV |  | x | 36 | 6 | U2-theraphotoxin-Cg1a | *Chilobrachys guangxiensis* | huwentoxin-1 | 74.3 | 4.00E-19 |
| U4-theraphotoxin-Pf7 | EVVLDSEQNRDCTKLLGGCKTDAECCPHLGCKVMCSQ |  | x | 37 | 6 | U18-theraphotoxin-Cg1a | *Chilobrachys guangxiensis* | huwentoxin-1 | 81 | 1.60E-10 |
| U4-theraphotoxin-Pf8 | DCTKLLGGCKTDAECCPHLGCRKKWPYHCGWDGPSDK | x |  | 37 | 6 | U18-theraphotoxin-Cg1a | *Chilobrachys guangxiensis* | huwentoxin-1 | 87.9 | 1.10E-23 |
| U4-theraphotoxin-Pf9 | ECRQFWGWCSRDSDCCKHLSCKRKWPNICLWDGTFTK | x |  | 37 | 6 | U28-theraphotoxin-Cg1a | *Chilobrachys guangxiensis* | huwentoxin-1 | 77.4 | 1.90E-16 |
| U4-theraphotoxin-Pf10 | EMFSLIAEGVCWCVDKTGRVLTNHQGHNIMLTFEIQS |  | x | 37 | 2 | U21-theraphotoxin-Cg1c | *Chilobrachys guangxiensis* | huwentoxin-1 | 68.2 | 1.10E-05 |
| U4-theraphotoxin-Pf11 | KKECSQLLGSCTKDSDCCSPFSCTPKWPRYCSWHSIFQI | x |  | 39 | 6 | U18-theraphotoxin-Cg1a | *Chilobrachys guangxiensis* | huwentoxin-1 | 52.8 | 2.20E-10 |
| U4-theraphotoxin-Pf12 | GCKTDAECCPHLGCRMGKFEAHWNTTCTTRSAPDSKSPS |  | x | 39 | 5 | U18-theraphotoxin-Cg1a | *Chilobrachys guangxiensis* | huwentoxin-1 | 80 | 2.00E-04 |
| U4-theraphotoxin-Pf13 | ECRYWLGGCEKTSDCCEHLSCSPKHGWCVWDWTF | x | x | 34 | 6 | U5-theraphotoxin-Hhn1a | *Haplopelma hainanum* | huwentoxin-1 | 88.9 | 2.00E-27 |
| U4-theraphotoxin-Pf14 | DCKQLFGTCKKDEECCEHLGCNKKYGWCGWDGTFGR | x |  | 36 | 6 | U1-theraphotoxin-Hhn1a | *Haplopelma hainanum* | huwentoxin-1 | 82.9 | 1.50E-21 |
| U4-theraphotoxin-Pf15 | ECRWYLGACKKDSDCCKHLQCHSYVGWCIWDGTQGQ | x |  | 36 | 6 | omega-theraphotoxin-Hhn1c | *Haplopelma hainanum* | huwentoxin-1 | 78.8 | 1.10E-19 |
| U4-theraphotoxin-Pf16 | ECRYWLGGCEKTSDCCEHLSCSPKHGWCVWDWTFRK | x |  | 36 | 6 | U5-theraphotoxin-Hhn1a | *Haplopelma hainanum* | huwentoxin-1 | 88.9 | 2.00E-27 |
| U4-theraphotoxin-Pf17 | MRCFSAMFSAEDQLSPQEKGMSDCISLYDSFFYHNLL |  | x | 37 | 2 | U5-theraphotoxin-Hhn1a | *Haplopelma hainanum* | huwentoxin-1 | 75 | 3.10E-04 |
| U4-theraphotoxin-Pf18 | LCKSDDDCCPHLMCYKYGWCGWDGSVGGSASLLLPNC |  | x | 37 | 6 | U1-theraphotoxin-Hhn1a | *Haplopelma hainanum* | huwentoxin-1 | 59.3 | 2.80E-06 |
| U4-theraphotoxin-Pf19 | FSAEDQLSPQERECRYWLGGCEKTSDCCEHLSCSPSVC |  | x | 38 | 6 | U5-theraphotoxin-Hhn1a | *Haplopelma hainanum* | huwentoxin-1 | 80 | 3.90E-11 |
| U4-theraphotoxin-Pf20 | ECRYFWGECNDKDLVCCDYLVCKYKWPLSYNICVWNRTFPG | x |  | 41 | 6 | U7-theraphotoxin-Hhn1e | *Haplopelma hainanum* | huwentoxin-1 | 77.5 | 2.30E-26 |
| U4-theraphotoxin-Pf21 | NCAKEGELCGWGSRCCHDLYCPAAVVAYCEP | x |  | 31 | 6 | tau-theraphotoxin-Hs1a | *Haplopelma schmidti* | huwentoxin-1 | 73.3 | 1.80E-13 |
| U4-theraphotoxin-Pf22 | CYASEVEELNLQDEDCNIYSDSCAGSVCRPFSWM |  | x | 34 | 4 | U5-theraphotoxin-Hs1b 2 | *Haplopelma schmidti* | huwentoxin-1 | 31.9 | 4.60E+00 |
| U4-theraphotoxin-Pf23 | QEYVPETENCAKEGELCGWGSRCCHDLYCPAALSDM | x | x | 36 | 5 | tau-theraphotoxin-Hs1a | *Haplopelma schmidti* | huwentoxin-1 | 76.5 | 2.10E-20 |
| U4-theraphotoxin-Pf24 | ESERASACAKEGEVCGWGKRCCDLDNYYCPAGIVPFC |  | x | 37 | 6 | tau-theraphotoxin-Hs1a | *Haplopelma schmidti* | huwentoxin-1 | 73.3 | 3.70E-16 |
| U4-theraphotoxin-Pf25 | ECRYFWGQCGGQEGNCCAHLVCRRKWPNICIWDLTV |  | x | 36 | 6 | kappa-theraphotoxin-Hm2a | *Heteroscodra maculata* | huwentoxin-1 | 61.1 | 1.60E-13 |
| U4-theraphotoxin-Pf26 | ECRYFWGQCGGQEGNCYFVKLNFSACNCCKSQTLPLH |  | x | 37 | 6 | kappa-theraphotoxin-Hm2a | *Heteroscodra maculata* | huwentoxin-1 | 88.9 | 2.60E-01 |
| U4-theraphotoxin-Pf27 | WYLGACKKDSDCCKHLQCHSYWEWCIWDGKVTF | x |  | 33 | 5 | tau-theraphotoxin-Pc1c | *Psalmopoeus cambridgei* | huwentoxin-1 | 82.8 | 5.40E-23 |
| U4-theraphotoxin-Pf28 | MCHNKEFMGLCKSDDDCCPHLMCYKYGWCGWDG |  | x | 33 | 6 | tau-theraphotoxin-Pc1c | *Psalmopoeus cambridgei* | huwentoxin-1 | 55.2 | 1.50E-06 |
| U4-theraphotoxin-Pf29 | CSRYFLGGCTEHSDCCEHLSCKMGLNYCAWDGTF |  | x | 34 | 6 | tau-theraphotoxin-Pc1b | *Psalmopoeus cambridgei* | huwentoxin-1 | 84.4 | 4.30E-20 |
| U4-theraphotoxin-Pf30 | GGCTEHSDCCEHLSCKMESQYKLFTMHCSICPEN |  | x | 34 | 6 | tau-theraphotoxin-Pc1b | *Psalmopoeus cambridgei* | huwentoxin-1 | 82.4 | 2.60E-06 |
| U4-theraphotoxin-Pf31 | ECRWYLGACKKDSDCCKHLQCHSYWEWCIWDGTIS | x | x | 35 | 6 | tau-theraphotoxin-Pc1c | *Psalmopoeus cambridgei* | huwentoxin-1 | 81.8 | 3.10E-25 |
| U4-theraphotoxin-Pf32 | AGCRYFLGGCTEHSDCCEHLSCKMGLNYCAWDGTF | x | x | 35 | 6 | tau-theraphotoxin-Pc1b | *Psalmopoeus cambridgei* | huwentoxin-1 | 84.8 | 1.60E-21 |
| U4-theraphotoxin-Pf33 | IFECSLSCDIKKEGKACKGKGEKKCGGGWRCKMNFCLKF | x | x | 39 | 6 | U3-theraphotoxin-Cg1b | *Chilobrachys guangxiensis* | huwentoxin-2 | 84.6 | 2.80E-25 |
| U4-theraphotoxin-Pf34 | IFGCTLSCDIKKEGKACKGKGEKKCGGGWRCKFNFCIRF | x |  | 39 | 6 | U3-theraphotoxin-Cg1b | *Chilobrachys guangxiensis* | huwentoxin-2 | 76.9 | 3.10E-22 |
| U4-theraphotoxin-Pf35 | CIGEGVPCDENDPRCCSKLECLKPKGYGWWYKSYYCYKKKSS | x | x | 42 | 6 | omega-theraphotoxin-Bs2a | *Brachypelma smithi* | huwentoxin-1 | 78 | 1.20E-25 |
| U7-theraphotoxin-Pf1 | CWGANVPCENEKSPCCRGLSCEKTFGYGWWYGSPFCVRKK | x |  | 41 | 6 | U26-theraphotoxin-Cg1a | *Chilobrachys guangxiensis* | Jztx-56 | 86.8 | 2.30E-27 |
| U9-theraphotoxin-Pf1 | SESYRASSCILVGDPCESTCDCCGWTTSCRHSKSAF | x | x | 36 | 6 | U32-theraphotoxin-Cg1a | *Chilobrachys guangxiensis* | Tx2 | 67.6 | 2.90E-12 |
| U9-theraphotoxin-Pf2 | PDADLIETYMGSESYRASSCILVGDPCESTCDCCGW |  | x | 36 | 5 | U32-theraphotoxin-Cg1a | *Chilobrachys guangxiensis* | Tx2 | 63.5 | 9.70E-19 |
| U9-theraphotoxin-Pf3 | GSESYRASSCILVGDPCESTCDCCGWTTSCRHSKSARS |  | x | 38 | 6 | U32-theraphotoxin-Cg1a | *Chilobrachys guangxiensis* | Tx2 | 64.1 | 8.30E-14 |
| U9-theraphotoxin-Pf4 | YCERYEYPDADLIETYMGSESYRASSCILVGDPCESTCD |  | x | 39 | 4 | U32-theraphotoxin-Cg1a | *Chilobrachys guangxiensis* | Tx2 | 54.1 | 1.70E-07 |
| U9-theraphotoxin-Pf5 | ASSCILVGDPCESTCDCCGWTTSCRHSKSAGRIVLQPIQNPA |  | x | 42 | 6 | U32-theraphotoxin-Cg1a | *Chilobrachys guangxiensis* | Tx2 | 75.9 | 3.30E-12 |
| U9-theraphotoxin-Pf6 | ASSCILVGDPCESTCDCCGWTTSCRHSKSAGEKVCKEGSKIKGLNTIMKGVAAAKKANCVHKHY | x |  | 64 | 8 | U32-theraphotoxin-Cg1a | *Chilobrachys guangxiensis* | Tx2 | 68.9 | 3.60E-27 |
| U1-ctenitoxin-Vf1 | KENCGTTGHDCEDVKCCEGYRCLYTARTGRLWMCL | x | x | 35 | 6 | U6-agatoxin-Ao1a | *Agelena orientalis* | _ | 48 | 7.50E-01 |
| U1-ctenitoxin-Vf2 | GYCAEKGVKCHNIHCCENLRCKCNDDRSSCVCRKNKVS | x |  | 38 | 8 | U4-agatoxin-Ao1a | *Agelena orientalis* | _ | 76.5 | 1.70E-16 |
| U1-ctenitoxin-Vf3 | FCVKTGDSCSGLCQCCDPEADCECRGEFIPGKSPCSCKDRGNVKVCKWKQEMCRINRPKNC | x |  | 61 | 12 | U9-agatoxin-Ao1a | *Agelena orientalis* | _ | 39.6 | 4.10E-05 |
| U3-ctenitoxin-Vf1 | EKICADKGDRCDKGLKCCKGPCVVSSLSFNIMISKCIR | x |  | 38 | 6 | purotoxin-1 | *Geolycosa sp* | _ | 57.9 | 4.60E+00 |
| U2-ctenitoxin-Vf1 | STKCTKRNHDCTGDRHRCCRGKMFKDVCMCFYKEGNETAR | x |  | 40 | 6 | CSTX-14 | *Cupiennius salei* | CSTX | 82.8 | 1.20E-17 |
| U5-ctenitoxin-Vf1 | EKCRKTCDCCERPNAICECTAEFWVGKSYCYCKEGELINCQLKKQGCRNQASK | x |  | 53 | 10 | U13-lycotoxin-Ls1b | *Lycosa singoriensis* | spider agouti | 34.5 | 3.90E-04 |
| U7-ctenitoxin-Vf1 | EDKLGRCTLGDCDPSKSHCQCCGDFTYCGCFWDISWLGNCRCRPGTRVSCFEKR | x |  | 54 | 10 | mu-ctenitoxin-Pn1a | *Phoneutria nigriventer* | Tx1 | 51.5 | 9.00E-07 |
| U7-ctenitoxin-Vf2 | CRKTCDCCERPNAICECTAEFWVGKSYCYCKER | x |  | 33 | 8 | delta-ctenitoxin-Pr2d | *Phoneutria reidyi* | Tx2 | 44.8 | 1.40E+00 |
| U7-ctenitoxin-Vf3 | GCAKRGKSCDHVKCCPNSACRCNGFRTNCRCGSPGLFTMIG | x |  | 41 | 8 | omega-agatoxin-Aa5a | *Agelenopsis aperta* | Tx3 | 60 | 7.10E-04 |
| U7-ctenitoxin-Vf4 | CIDVYETCNKGIPCCEDRPCKCNIVMDNCICKKTIAELFR | x |  | 40 | 8 | kappa-ctenitoxin-Pn1a | *Phoneutria nigriventer* | Tx3 | 72.5 | 2.30E-18 |
| U7-ctenitoxin-Vf5 | APKCIDVYETCNKGIPCCEDRPCRCNIVMDNCICKKTIAELFGGK | x |  | 45 | 8 | kappa-ctenitoxin-Pn1a | *Phoneutria nigriventer* | Tx3 | 70.5 | 1.20E-18 |
| U7-ctenitoxin-Vf6 | CYETCCEDRPCRCNIVMDNCICKKTIAELFGGNNYYTRQDEVTCLL | x |  | 46 | 8 | kappa-ctenitoxin-Pn1a | *Phoneutria nigriventer* | Tx3 | 76.7 | 4.10E-15 |
| U7-ctenitoxin-Vf7 | APKCIDVYETCNKGIPCCEDRPCKCNIVMDNCICKNSKDVSSKNCSAENNFYTRQDE | x |  | 57 | 9 | kappa-ctenitoxin-Pn1a | *Phoneutria nigriventer* | Tx3 | 70.6 | 2.60E-13 |
| U7-ctenitoxin-Vf8 | APKCIDVYETCNKGNTLAVKTDLANANIVMDNCICKKTIAELFGGNNFYTRQDEQYWHRLVNIHNHLKLK | x |  | 70 | 4 | kappa-ctenitoxin-Pn1a | *Phoneutria nigriventer* | Tx3 | 52.4 | 3.60E-08 |
| U7-ctenitoxin-Vf9 | APKCIDVYETCNKGIPCCEDRPCRCNIVMDNCICKKTIAELFGGSYLPSLNSVQLDLKEKSLQIIIMSSKVFLFILLLLSLQFLEQML | x |  | 88 | 8 | kappa-ctenitoxin-Pn1a | *Phoneutria nigriventer* | Tx3 | 69 | 5.00E-15 |
| U8-ctenitoxin-Vf1 | YKKCNQTGKDCSNDCDCCNKWTCKCPIWGLFGCSCVFGDSMV | x |  | 42 | 9 | U18-ctenitoxin-Pn1a | *Phoneutria nigriventer* | type II/III omega-agatoxin | 77.5 | 8.90E-22 |
| U8-ctenitoxin-Vf2 | YKKCNQTGKDCSNDCDCCNKWTYCKCPIWGLFGCSCVFGDSMVCVRKREQCKDPAVMDFQKEAAFLKEEECT | x |  | 72 | 12 | U18-ctenitoxin-Pn1a | *Phoneutria nigriventer* | type II/III omega-agatoxin | 79.5 | 4.20E-26 |
| U8-ctenitoxin-Vf3 | SCKKREEACVTDCDCCGGFYICHYPAVIKYLGVGGSCIYGNKYDCER | x |  | 47 | 8 | U7-ctenitoxin-Pr1a | *Phoneutria reidyi* | type II/III omega-agatoxin | 49 | 3.30E-11 |
| U8-ctenitoxin-Vf4 | SCKKRAEACKTDCDCCGGFYKCHFPAGLKYFGFGGSCVYGNKYDCER | x |  | 47 | 8 | U7-ctenitoxin-Pr1a | *Phoneutria reidyi* | type II/III omega-agatoxin | 56.9 | 7.70E-15 |
| U9-ctenitoxin-Vf3 | CIPKHYECTHHKDECCKGHLFHYKCRCYKIAGDKGEESKR | x |  | 40 | 6 | U1-lycotoxin-Ls1hh | *Lycosa singoriensis* | U1-lycotoxin | 60 | 3.50E-15 |
| U9-ctenitoxin-Vf4 | QCIRYEHVCSFNKGECCTGLKCECYDRYIKGEKGEEKCWCIEKDVMYKKRGE | x |  | 52 | 8 | U6-lycotoxin-Ls1g | *Lycosa singoriensis* | U6-lycotoxin | 53.2 | 5.90E-14 |
| U9-ctenitoxin-Vf5 | QCIQYEHVCSFNIGKCCPGLKCECYDRYIKGEKGEEKCWCIEKDVMYKKRGE | x |  | 52 | 8 | U6-lycotoxin-Ls1g | *Lycosa singoriensis* | U6-lycotoxin | 53.2 | 2.50E-13 |
| U1-theritoxin-Lm1 | KQCIKKHYECTHDKRNCCVGKVFQYTCKCYDYTNSAGVVEPRCKCTKSILGALTDFGVNLWNRVTG | x |  | 66 | 8 | U1-ctenitoxin-Cs1a | *Cupiennius salei* | CSTX | 44 | 1.70E-12 |
| U3-theritoxin-Lm1 | DECTPLTHDCTHDRHSCCRGPTFKYKCDCLYPFDNSTSAWDQTELCFCVEPGVHHFLDEVMDKTIGIFG | x |  | 69 | 8 | purotoxin-2 | *Geolycosa sp.* | spider toxin CSTX | 54.1 | 2.50E-22 |
| U3-theritoxin-Lm2 | SECTPLTHDCTDDRHNCCRGPTFKYKCECLHPYINETNTWDEKELCFCVEPGVHHWFDEAVDKAGSFFW | x |  | 69 | 8 | purotoxin-2 | *Geolycosa sp.* | spider toxin CSTX | 46.8 | 1.00E-16 |
| U3-theritoxin-Lm3 | DECTPLTHDCTHDRHSCCRGPTFEYRCECLYPFDESTGTWDTKEMCFCIEPGLHHFFDQHLEKA | x |  | 64 | 8 | purotoxin-2 | *Geolycosa sp.* | spider toxin CSTX | 51.6 | 2.00E-19 |
| U3-theritoxin-Lm4 | SNECTPLTHDCTDDRHNCCRGPTFKYKCECLHPYINETNTWDEKELCFCVEPGVHHWFDEAVDKAGSFLG | x |  | 70 | 8 | purotoxin-2 | *Geolycosa sp.* | spider toxin CSTX | 45.5 | 7.60E-17 |
