# Supplementary material for: Peptidomic and transcriptomic profiling of four distinct spider venoms
Source: PLoS One. 2017 Mar 17;12(3):e0172966. doi: 10.1371/journal.pone.0172966 (PMC5357004; doi:10.1371/journal.pone.0172966)
Supplement: S2 Table — (DOCX) [file pone.0172966.s002.docx]

| Specie | RPKM |
| --- | --- |
|  | Agatoxins |
| *H. davidbowie* | 12'109 |
| *V. fasciatus* | 5'814 |
|  | CSTX |
| *H. davidbowie* | 1'819 |
| *V. fasciatus* | 53 |
| *L. mactans* | 5'417 |
|  | Huwentoxins |
| *H. davidbowie* | 13'326 |
| *P. formosa* | 208'549 |
|  | Phrixotoxins |
| *H. davidbowie* | 22'158 |
|  | Tx |
| *H. davidbowie* | 13'161 |
| *P. formosa* | 53'671 |
| *V. fasciatus* | 15'592 |
|  | Lycotoxins |
| *H. davidbowie* | 2'848 |
| *V. fasciatus* | 602 |
|  | LiTx3 |
| *H. davidbowie* | 24 |
|  | Jztx-56 |
| *P. formosa* | 7'731 |
|  | spider agouti |
| *V. fasciatus* | 4'518 |
|  | Total |
| *H. davidbowie* | 43'287 |
| *P. formosa* | 269'951 |
| *V. fasciatus* | 26'579 |
| *L. mactans* | 5'417 |
