# Supplementary material for: Peptidomic and transcriptomic profiling of four distinct spider venoms
Source: PLoS One. 2017 Mar 17;12(3):e0172966. doi: 10.1371/journal.pone.0172966 (PMC5357004; doi:10.1371/journal.pone.0172966)
Supplement: S3 Table — (DOCX) [file pone.0172966.s003.docx]

| Proposed Name | Predicted/ Detected Mature Sequence | Retrieved by HMM | Detected by MS/MS | Mass [Da] | PTM mass [Da] | Length | Cys number | Complete | Rt [min] | RPKM | Match found in Uniprot | Specie | Family | % Identity | e-value | Uniprot Code |
| --- | --- | --- | --- | --- | --- | --- | --- | --- | --- | --- | --- | --- | --- | --- | --- | --- |
| U1-sparatoxin-Hdb1 | TEAFYMSIEDAALDTVMARDDDKKDCVGHMGWCAWTDSECC |  | x | 4’649.8786 | 32.0086 | 41 | 4 | x | 58.93 | 6’059 | mu-agatoxin-Aa1f | *Agelenopsis aperta* | beta/delta agatoxin | 52.4 | 7.70E-01 | P11062 |
| U1-sparatoxin-Hdb2 | MGWCAWTDSECCEGYRCSSPYPAAAAELPSGGLRGK |  | x | 3’809.6209 | _ | 36 | 4 | x | 61.28 | 4’825 | mu-agatoxin-Aa1f | *Agelenopsis aperta* | beta/delta agatoxin | 47.4 | 2.10E+00 | P11062 |
| U1-sparatoxin-Hdb3 | AKSLPEGAPCDGDKDDCQCYGKWHKCRCPWFWEDGPCRCAWGLKHTCITKLSCPNKGEWGLDWRSEEERSPC | x |  |  |  | 72 | 10 | x |  | 1’225 | omega-agatoxin-1A | *Agelenopsis aperta* | type I omega-agatoxin | 72.4 | 1.70E-45 | P15969 |
| U2-sparatoxin-Hdb1 | FCPAKSDIVCVWAQNKCCSDRDCPKGHLCCSENCGN | x |  |  |  | 36 | 8 | x |  | 293 | U7-agatoxin-Ao1a | *Agelena orientalis* | _ | 52.6 | 2.40E-03 | Q5Y4V9 |
| U3-sparatoxin-Hdb1 | ECIKQSEDCTNKRNGCCPSNEVFFESYCLCYLTASTRFVPKP | x |  |  |  | 42 | 6 | x |  | 1’819 | latartoxin-1a | *Lachsana tarabaevi* | CSTX | 48.5 | 4.80E-03 | B3EWF2 |
| U4-sparatoxin-Hdb2 | ERDCRKFMGLCKSDDDCCPHLMCYKYGWCGWDGS |  | x | 4’005.5709 | -2.0309 | 34 | 6 | x | 79.32 | 661 | beta-theraphotoxin-Cm2a | *Ceratogyrus marshalli* | huwentoxin-1 | 68.8 | 1.80E-10 | P84509 |
| U4-sparatoxin-Hdb6 | DLGDALFSAEDQQNLQERDCRKFMGLCKSDDDCCPHLMCYKYGWCG |  | x | 5’323.2209 | 15.9885 | 46 | 6 | x | 67.82 | 661 | beta-theraphotoxin-Cn2a | *Ceratogyrus marshalli* | huwentoxin-1 | 66.7 | 1.40E-09 | P84509 |
| U4-sparatoxin-Hdb13 | DCKWIFGSCETSDVCCEGWVCSKGLCKYKLWR | x |  |  |  | 32 | 6 | x |  | 1’978 | omega-sparatoxin-Hv1a | *Heteropoda venatoria* | huwentoxin-1 | 55.6 | 3.40E-07 | P61789 |
| U4-sparatoxin-Hdb14 | CIGHLDSCLTASCCPGYKCKCDSIDCLCV | x |  |  |  | 29 | 8 | x |  | 292 | U24-theraphotoxin-Cg1a | *Chilobrachys guangxiensis* | huwentoxin-1 | 100 | 2.70E+00 | B1P1H2 |
| U4-sparatoxin-Hdb15 | GDDKENCKYWFDSCETEGECCDNWTCHNGICKTIVKAVVRSHKTWSKV |  | x | 3’603.2900 | _ | 48 | 6 | x | 49.69 | 1’133 | omega-sparatoxin-Hv1b | *Heteropoda venatoria* | huwentoxin-1 | 55.6 | 6.50E-04 | P61790 |
| U4-sparatoxin-Hdb16 | GDDKENCKYWFDSCETEGECCDNWTCHNGICKIKIIL |  | x | 4’312.8209 | _ | 37 | 6 | x | 52.29 | 8’205 | omega-sparatoxin-Hv1b | *Heteropoda venatoria* | huwentoxin-1 | 55.6 | 3.20E-04 | P61790 |
| U4-sparatoxin-Hdb17 | MNLHSTMFCLHSCETEGECCDNWTCHNGICKIKIIL |  | x | 4’142.7709 | 15.9806 | 36 | 6 | x | 53.43 | 396 | omega-sparatoxin-Hv1b | *Heteropoda venatoria* | huwentoxin-1 | 60 | 5.90E-04 | P61790 |
| U5-sparatoxin-Hdb1 | DCVKDGGSCNNGETCCQGGKYKRICGCEAWNPGHCTCHDDCQVC | x | x | 4’695.7800 | _ | 44 | 10 | x | 63.75 | 24 | U2-sicaritoxin-Li1b | *Loxosceles intermedia* | LiTx3 | 37.3 | 2.00E-02 | P0DMD8 |
| U6-sparatoxin-Hdb11 | DCIGHMGWCAWTDSECCVGYRCKLWCRKCVPLVTRSTTAALCPDSSI | x |  |  |  | 47 | 8 | x |  | 74 | beta/kappa-theraphotoxin-Cg2a | *Chilobrachys guangxiensis* | phrixotoxin | 71.4 | 7.90E-04 | Q2PAY4 |
| U6-sparatoxin-Hdb12 | DCVGHMGWCAWTDSECCEGYRCKLWCRKI | x |  |  |  | 29 | 6 | x |  | 743 | beta/kappa-theraphotoxin-Cg2a | *Chilobrachys guangxiensis* | phrixotoxin | 73.9 | 9.30E-07 | Q2PAY4 |
| U6-sparatoxin-Hdb13 | DCIGWMGWCSGKELKCCEGHVCSLWCKKKLG | x |  |  |  | 31 | 6 | x |  | 1’310 | beta/kappa-theraphotoxin-Tp2a | *Thrixopelma pruriens* | phrixotoxin | 71.4 | 8.20E-09 | P83476 |
| U6-sparatoxin-Hdb14 | DCIGWMGWCSGKDKKCCKGNVCNLWCRYKADVLDLISLIR | x |  |  |  | 40 | 6 | x |  | 208 | kappa-theraphotoxin-Cg2b | *Chilobrachys guangxiensis* | phrixotoxin | 54.2 | 6.70E-06 | B1P1B6 |
| U6-sparatoxin-Hdb15 | DCNGWTAWCNNCCEDFVCNIWCSLKQALKE | x |  |  |  | 30 | 6 | x |  | 1’067 | beta-theraphotoxin-Gr1a | *Grammostola rosea* | phrixotoxin | 52.4 | 6.00E-03 | P85117 |
| U6-sparatoxin-Hdb16 | DDDCIGWMGLCSSSEKKCCEGYACEVWCKYD | x |  |  |  | 31 | 6 | x |  | 900 | kappa-theraphotoxin-Cg2b | *Chilobrachys guangxiensis* | phrixotoxin | 68 | 1.50E-08 | B1P1B6 |
| U6-sparatoxin-Hdb17 | DDDKKDCIGHMGWCAWTDSECCVGYRCKLWCRKIIDWLGD | x |  |  |  | 40 | 6 | x |  | 2’298 | beta/kappa-theraphotoxin-Cg2a | *Chilobrachys guangxiensis* | phrixotoxin | 73.9 | 4.80E-06 | Q2PAY4 |
| U6-sparatoxin-Hdb18 | DDDCIGWMGLCSSSEKKCCEGYACEVWCKYDLDGEKV | x |  |  |  | 37 | 6 | x |  | 1’955 | kappa-theraphotoxin-Cg2a | *Chilobrachys guangxiensis* | phrixotoxin | 65.5 | 7.50E-10 | P0C5X7 |
| U6-sparatoxin-Hdb19 | DDDKKDCVGHMGWCAWTDSECCVGYRCKLWCRKI |  | x | 4’009.7209 | _ | 34 | 6 | x | 46.70 | 1’371 | beta/kappa-theraphotoxin-Cg2a | *Chilobrachys guangxiensis* | phrixotoxin | 72.7 | 4.60E-06 | Q2PAY4 |
| U6-sparatoxin-Hdb20 | GDDEDCIGWMGWCSGKDKKCCKGNVCNLWCRYKADV |  | x | 4’101.7265 | 15.9913 | 36 | 6 | x | 43.61 | 5’662 | kappa-theraphotoxin-Cg2b | *Chilobrachys guangxiensis* | phrixotoxin | 54.2 | 6.00E-06 | B1P1B6 |
| U6-sparatoxin-Hdb21 | GDDQTCIGWMGWCSGKNIGCCEGYKCELWCKYA |  | x | 3’707.4982 | _ | 33 | 6 | x | 57.13 | 429 | kappa-theraphotoxin-Cg2a | *Chilobrachys guangxiensis* | phrixotoxin | 63 | 2.30E-07 | P0C5X7 |
| U6-sparatoxin-Hdb22 | MARDDDKKDCVGHMGWCAWTDSECCVGYRCKLMC |  | x | 3’914.5700 | 32.0012 | 34 | 6 | x | 59.88 | 886 | beta/kappa-theraphotoxin-Cg2a | *Chilobrachys guangxiensis* | phrixotoxin | 69.6 | 4.50E-03 | Q2PAY4 |
| U6-sparatoxin-Hdb23 | EDDKDCNGWTAWCNNCCEDFVCNMWCSLKQALKE |  | x | 4’001.5809 | _ | 34 | 6 | x | 79.21 | 5’255 | beta-theraphotoxin-Gr1a | *Grammostola rosea* | phrixotoxin | 52.4 | 4.10E-03 | P85117 |
| U7-sparatoxin-Hdb1 | KGPCPLYYRINDCCKQSDCREGSTCCKLQCGNACQR | x |  |  |  | 36 | 8 | x |  | 509 | U20-lycotoxin-Ls1d | *Lycosa singoriensis* | spider wap-2 | 67.7 | 3.40E-09 | B6DCY1 |
| U7-sparatoxin-Hdb2 | PVEDECPLKYKINDCCAQSDCRAGDICCSEPCGYVCRR | x |  |  |  | 38 | 8 | x |  | 765 | U20-lycotoxin-Ls1d | *Lycosa singoriensis* | spider wap-2 | 72.7 | 2.50E-15 | B6DCY1 |
| U7-sparatoxin-Hdb3 | KGPCRMDYKINDCCKQSDCPSQSTCCKLPCRHVCQRESR | x |  |  |  | 39 | 8 | x |  | 7’526 | U20-lycotoxin-Ls1d | *Lycosa singoriensis* | spider wap-2 | 57.1 | 1.10E-06 | B6DCY1 |
| U7-sparatoxin-Hdb4 | FCPGFTDEVCPFDYKINDCCAQSDCPSYAICCVQPCGNVCRRKADKPI | x |  |  |  | 48 | 9 |  |  | 2’073 | U20-lycotoxin-Ls1d | *Lycosa singoriensis* | spider wap-2 | 56.3 | 8.40E-14 | B6DCY1 |
| U7-sparatoxin-Hdb5 | PCGNVCRPKFDKPIGTPFVDGTECEIGHVFPKAWYQRDSLQKL | x |  |  |  | 43 | 3 |  |  | 1’422 | U20-lycotoxin-Ls1d | *Lycosa singoriensis* | spider wap-2 | 68.6 | 3.20E-14 | B6DCY1 |
| U7-sparatoxin-Hdb6 | FCPDFTDKVCPVSYKINDCCAQSDCPSEAICCVQPCGNVCRPKSDKPIGTPFVDGTECETGHVFPKAWYQRWWEWITSGRMQI | x |  |  |  | 83 | 10 | x |  | 14’806 | U20-lycotoxin-Ls1b | *Lycosa singoriensis* | spider wap-2 | 64.4 | 6.20E-37 | B6DD61 |
| U8-sparatoxin-Hdb1 | GQCADVWKRCGNGVECCCNRSCICNVTRTKCKCHRFHEVSHGYTAECLV | x |  |  |  | 49 | 10 | x |  | 12’762 | kappa-ctenitoxin-Pn1a | *Phoneutria nigriventer* | Tx3 | 44.4 | 3.30E-04 | O76200 |
| U8-sparatoxin-Hdb2 | AIEVCGQLYKECSKRFKCCENR | x |  |  |  | 22 | 4 | x |  | 53 | omega-ctenitoxin-Pr1a | *Phoneutria reidyi* | Tx3 | 50 | 9.00E+00 | P83911 |
| U8-sparatoxin-Hdb3 | AIEVCGQLYKECSKRFKCCENRPCKCNKRGER | x |  |  |  | 32 | 6 | x |  | 346 | omega-ctenitoxin-Pr1a | *Phoneutria reidyi* | Tx3 | 55.6 | 6.60E-05 | P83911 |
| U9-sparatoxin-Hdb1 | QCIKLEGECTKNKDNCCAEHRCRCYDKYVNGIKTEVR | x |  |  |  | 37 | 6 | x |  | 865 | U6-lycotoxin-Ls1f | *Lycosa singoriensis* | U6-lycotoxin | 51.6 | 5.20E-05 | B6DCV6 |
| U9-sparatoxin-Hdb2 | QCIKLEGECTKNKDNCCAEHRCRCYDKYVNWNKNR | x |  |  |  | 35 | 6 | x |  | 61 | U6-lycotoxin-Ls1f | *Lycosa singoriensis* | U6-lycotoxin | 62.5 | 3.60E-04 | B6DCV6 |
| U9-sparatoxin-Hdb3 | QCIKLEGECTKNKDNCCAEHRCRCYDKYVNGIKTEVRCWCFEKDVTYKPTFEIK | x |  |  |  | 54 | 8 | x |  | 960 | U6-lycotoxin-Ls1e | *Lycosa singoriensis* | U6-lycotoxin | 55.3 | 9.90E-17 | B6DCV5 |
| U9-sparatoxin-Hdb4 | QCTPQEHRCFKGAPKCCGGFDCQCYTPIVNGVKEEPTCWCNEPNVIYEYAFKAQY | x |  |  |  | 55 | 8 | x |  | 962 | U6-lycotoxin-Ls1g | *Lycosa singoriensis* | U6-lycotoxin | 40.4 | 9.80E-08 | B6DCV8 |
| U10-sparatoxin-Hdb1 | QQYKTACPCDEGMFCSTI |  | x | 2’024.8221 | _ | 18 | 3 |  | 40.38 | 2’369 | U8-theraphotoxin-Hhn1g | *Haplopelma hainanum* | AVIT | 57.9 | 9.30E-03 | D2Y2E7 |
| PLA2-like peptide Hdb1 | FDLVTGLDPSDYIPYGNWCGYGGEGKPVDRIDGCC |  | x | 3’823.6582 | 41.9974 | 35 | 3 |  | 62.24 | 47 | PLA2 | *Scolopendra viridis* | PLA2 | 64.9 | 7.70E-12 | C1JAR9 |
| CRISP-like peptide Hdb1 | ENGEMYKAEDSFRHPALAMIACACAQVYNYECY |  | x | 3’806.6077 | 15.9974 | 33 | 3 |  | 60.13 | 51 | venom allergen 5 | *Tityus serrulatus* | CRISP | 31.7 | 5.70E-02 | P85840 |
| CRISP-like peptide Hdb2 | VAQKLAETCHYDHDCNPCRRVQ |  | x | 2’673.2209 | _ | 22 | 3 |  | 28.07 | 4’006 | CRISP/Allergen/PR-1 | *Trittame loki* | CRISP | 64.5 | 2.20E-09 | W4VS53 |
| CRISP-like peptide Hdb3 | VGMGQKEGNSYTSYTVCNYGPGGNVENGEMYKAGKTCSACP |  | x | 4’267.8209 | 16.0024 | 41 | 3 |  | 57.16 | 10’308 | CRISP/Allergen/PR-1 | *Trittame loki* | CRISP | 57.5 | 1.00E-08 | W4VS53 |
| CRISP-like peptide Hdb4 | SVAQKLAETCNYGHDCNPCRRVQSFSVGQNIGKVTGLGSPPSSADWRSFIKNLYDE |  | x | 6’128.9300 | _ | 56 | 3 |  | 58.86 | 10’872 | CRISP/Allergen/PR-1 | *Trittame loki* | CRISP | 47.7 | 8.70E-11 | W4VS53 |
