# Supplementary material for: Peptidomic and transcriptomic profiling of four distinct spider venoms
Source: PLoS One. 2017 Mar 17;12(3):e0172966. doi: 10.1371/journal.pone.0172966 (PMC5357004; doi:10.1371/journal.pone.0172966)
Supplement: S4 Table — (DOCX) [file pone.0172966.s004.docx]

| Proposed Name | Predicted/ Detected Mature Sequence | Retrieved by HMM | Detected by MS/MS | Mass [Da] | PTM mass [Da] | Length | Cys number | Complete | Rt [min] | RPKM | Match found in Uniprot | Specie | Family | % Identity | e-value | Uniprot Code |
| --- | --- | --- | --- | --- | --- | --- | --- | --- | --- | --- | --- | --- | --- | --- | --- | --- |
| U1-theraphotoxin-Pf1 | EIQECGHLHEKCNPGPPSTNTCCRGLQCRYGSCLVQV | x | x | 4’059.8209 | _ | 37 | 6 | x | 36.65 | 6’440 | U27-theraphotoxin-Cg1a | *Chilobrachys guangxiensis* | _ | 71.4 | 5.80E-15 | P0C201 |
| U1-theraphotoxin-Pf2 | CLPAGSACSGPLQKIPCCGTCSRKKCT | x |  |  |  | 27 | 6 | x |  | 56 | omega-theraphotoxin-Hs1a | *Haplopelma schmidti* | _ | 66.7 | 4.20E-08 | P68424 |
| U1-theraphotoxin-Pf3 | RCLPAGSACSGPIQKIPCCGTCSRKKCT | x | x | 2’868.3523 | _ | 28 | 6 | x | 29.81 | 2’418 | omega-theraphotoxin-Hs1a | *Haplopelma schmidti* | _ | 66.7 | 7.60E-09 | P68424 |
| U1-theraphotoxin-Pf4 | GCLKEGKWCPKSAPCCRPLVCKGPSIKQKKCT | x | x | 3’475.76 | _ | 32 | 6 | x | 27.09 | 401 | U12-theraphotoxin-Hs1a | *Haplopelma schmidti* | _ | 87.1 | 1.10E-19 | B3FIU2 |
| U1-theraphotoxin-Pf5 | KGCIKEGKWCPKSAPCCRPLVCKGPSIKQKKCT | x |  |  |  | 33 | 6 | x |  | 580 | U12-theraphotoxin-Hs1a | *Haplopelma schmidti* | _ | 87.1 | 9.00E-20 | B3FIU2 |
| U1-theraphotoxin-Pf6 | GCLKEGKWCPKSAPCCRPLVCKGPSIKQKKCTKP | x |  |  |  | 34 | 6 | x |  | 6’010 | U12-theraphotoxin-Hs1a | *Haplopelma schmidti* | _ | 87.1 | 1.40E-19 | B3FIU2 |
| U1-theraphotoxin-Pf7 | CLKEGKWCPKSAPCCRPLVCKGPSNKAEESCTINHLNSKA | x |  |  |  | 40 | 6 | x |  | 255 | U12-theraphotoxin-Hs1a | *Haplopelma schmidti* | _ | 84.6 | 3.70E-12 | B3FIU2 |
| U10-theraphotoxin-Pf1 | CGSQADCSEGSCCAGPSFAKNCRRYGDEGVQCEPWNKYEEYSTGCPCKENMICSAINRCQKA | x |  |  |  | 62 | 10 | x |  | 503 | U8-theraphotoxin-Hhn1f | *Haplopelma hainanum* | AVIT | 74.2 | 1.70E-36 | D2Y2E6 |
| U2-theraphotoxin-Pf1 | SCIKEWQICKNDCECCGMSTLCKSSWIDG | x |  |  |  | 31 | 6 | x |  | 226 | hainantoxin-XV-5 | *Haplopelma hainanum* | _ | 75.9 | 5.40E-16 | D2Y2D9 |
| U2-theraphotoxin-Pf2 | SCIKEWQICKNDCECCGMSTLCKSSWIDGREIKLCRNEGGKLKKVLHFIQKSVSKIKSCKK | x |  |  |  | 61 | 8 | x |  | 1’588 | hainantoxin-XV-4 | *Haplopelma hainanum* | _ | 86.9 | 4.30E-42 | D2Y2D8 |
| U2-theraphotoxin-Pf3 | ACSKQIGEKCSGNCDCCGSTVVCGSVYVGGKEEKFCSDKSSNNAALNTAGKGINAVSNMFSFCWG | x |  |  |  | 65 | 8 | x |  | 12’321 | hainantoxin-XVIII-6 | *Haplopelma hainanum* | _ | 66.7 | 6.30E-29 | D2Y2P0 |
| U3-theraphotoxin-Pf1 | NYCEYISRPGDQREYRSCVYTCTGNGCNAANSLTPAPT |  | x | 4’174.8209 | _ | 38 | 4 | x | 32.34 | 367 | omega-scoloptoxin-Ssm1a | *Scolopendra mutilans* | _ | 25.6 | 2.20E+00 | I6R1R5 |
| CRISP-like peptide Pf1 | NMLEMVWDDELAQIAQKWAYSCPSSKSDCDLCRMVD |  | x | 4’196.8209 | 15.9958 | 36 | 3 |  | 46.73 | 147 | venom allergen 5 | *Lycosa singoriensis* | CRISP | 62.6 | 1.50E-41 | A9QQ26 |
| CRISP-like peptide Pf2 | AANMLQMVWDDELAAVAQKHASQCTFEHDCDSSK |  | x | 3708.6209 | _ | 34 | 2 | x | 59.68 | 12’580 | CRISP/Allergen/PR-1 | *Trittame loki* | CRISP | 64.3 | 4.70E-18 | W4VS53 |
| CRISP-like peptide Pf3 | CNYGPGGNTEELPIYKKGNPCTSCPINSCCGTSCKEEQA |  | x | 4’095.7209 | _ | 39 | 6 | x | 51.56 | 13’815 | CRISP/Allergen/PR-1 | *Trittame loki* | CRISP | 60 | 2.70E-19 | W4VS53 |
| U4-theraphotoxin-Pf1 | DCRKFMGLCKSDDDCCPHLMCYKYGWCGWDGSV | x | x | 3’821.5382 | _ | 33 | 6 | x | 49.71 | 6’193 | beta-theraphotoxin-Cm2a | *Ceratogyrus marshalli* | huwentoxin-1 | 66.7 | 1.20E-09 | P84509 |
| U4-theraphotoxin-Pf2 | GDSENENLQERDCRKFMGLCKSDDDCCPHLMCYKYGCVDGWQC |  | x | 5’008.9847 | 15.9947 | 43 | 7 |  | 36.88 | 4’268 | beta-theraphotoxin-Cm2a | *Ceratogyrus marshalli* | huwentoxin-1 | 56.3 | 5.50E-06 | P60590 |
| U4-theraphotoxin-Pf3 | ECRWLFGGCTKDADCCKHLGCTRSYPQYCGWDLTV | x | x | 4’014.7209 | _ | 35 | 6 | x | 49.14 | 5’088 | U21-theraphotoxin-Cg1a 3 | *Chilobrachys guangxiensis* | huwentoxin-1 | 75.7 | 4.60E-19 | B1P1G3 |
| U4-theraphotoxin-Pf4 | ECRWLFGGCTKDADCCKHLGCRRSYPQYCGWDLTV |  | x | 4’051.7709 | -18.0136 | 35 | 6 | x | 49.60 | 3’091 | U21-theraphotoxin-Cg1a | *Chilobrachys guangxiensis* | huwentoxin-1 | 78.4 | 7.60E-21 | B1P1G3 |
| U4-theraphotoxin-Pf5 | AECRWMFGSCKEDSDCCKHLGCRRKAPQYCAWDGTV | x | x | 4’139.7709 | _ | 36 | 6 | x | 34.43 | 12’253 | U2-theraphotoxin-Cg1a | *Chilobrachys guangxiensis* | huwentoxin-1 | 74.3 | 2.40E-18 | B1P1A2 |
| U4-theraphotoxin-Pf6 | AECRWMFGSCKEDSDCCKHLGCRRDAPQYCAWDGTV |  | x | 4’126.7054 | _ | 36 | 6 | x | 38.86 | 3’922 | U2-theraphotoxin-Cg1a | *Chilobrachys guangxiensis* | huwentoxin-1 | 74.3 | 4.00E-19 | B1P1A2 |
| U4-theraphotoxin-Pf7 | EVVLDSEQNRDCTKLLGGCKTDAECCPHLGCKVMCSQ |  | x | 4’028.7709 | 15.9887 | 37 | 6 | x | 48.71 | 11’583 | U18-theraphotoxin-Cg1a | *Chilobrachys guangxiensis* | huwentoxin-1 | 81 | 1.60E-10 | B1P1F5 |
| U4-theraphotoxin-Pf8 | DCTKLLGGCKTDAECCPHLGCRKKWPYHCGWDGPSDK | x |  |  |  | 37 | 6 | x |  | 15’040 | U18-theraphotoxin-Cg1a | *Chilobrachys guangxiensis* | huwentoxin-1 | 87.9 | 1.10E-23 | B1P1F5 |
| U4-theraphotoxin-Pf9 | ECRQFWGWCSRDSDCCKHLSCKRKWPNICLWDGTFTK | x |  |  |  | 37 | 6 | x |  | 10’440 | U28-theraphotoxin-Cg1a | *Chilobrachys guangxiensis* | huwentoxin-1 | 77.4 | 1.90E-16 | B1P1H9 |
| U4-theraphotoxin-Pf10 | EMFSLIAEGVCWCVDKTGRVLTNHQGHNIMLTFEIQS |  | x | 4’207.0209 | _ | 37 | 2 |  | 61.63 | 4’908 | U21-theraphotoxin-Cg1c | *Chilobrachys guangxiensis* | huwentoxin-1 | 68.2 | 1.10E-05 | B1P1G5 |
| U4-theraphotoxin-Pf11 | KKECSQLLGSCTKDSDCCSPFSCTPKWPRYCSWHSIFQI | x |  |  |  | 39 | 6 | x |  | 7’771 | U18-theraphotoxin-Cg1a | *Chilobrachys guangxiensis* | huwentoxin-1 | 52.8 | 2.20E-10 | B1P1F5 |
| U4-theraphotoxin-Pf12 | GCKTDAECCPHLGCRMGKFEAHWNTTCTTRSAPDSKSPS |  | x | 4’212.8209 | _ | 39 | 5 |  | 36.32 | 383 | U18-theraphotoxin-Cg1a | *Chilobrachys guangxiensis* | huwentoxin-1 | 80 | 2.00E-04 | B1P1F5 |
| U4-theraphotoxin-Pf13 | ECRYWLGGCEKTSDCCEHLSCSPKHGWCVWDWTF | x | x | 4’051.6709 | _ | 34 | 6 | x | 54.54 | 4’486 | U5-theraphotoxin-Hhn1a | *Haplopelma hainanum* | huwentoxin-1 | 88.9 | 2.00E-27 | D2Y2C3 |
| U4-theraphotoxin-Pf14 | DCKQLFGTCKKDEECCEHLGCNKKYGWCGWDGTFGR | x |  |  |  | 36 | 6 | x |  | 3’210 | U1-theraphotoxin-Hhn1a | *Haplopelma hainanum* | huwentoxin-1 | 82.9 | 1.50E-21 | P0CH70 |
| U4-theraphotoxin-Pf15 | ECRWYLGACKKDSDCCKHLQCHSYVGWCIWDGTQGQ | x |  |  |  | 36 | 6 | x |  | 387 | omega-theraphotoxin-Hhn1c | *Haplopelma hainanum* | huwentoxin-1 | 78.8 | 1.10E-19 | D2Y2F1 |
| U4-theraphotoxin-Pf16 | ECRYWLGGCEKTSDCCEHLSCSPKHGWCVWDWTFRK | x |  |  |  | 36 | 6 | x |  | 8’099 | U5-theraphotoxin-Hhn1a | *Haplopelma hainanum* | huwentoxin-1 | 88.9 | 2.00E-27 | D2Y2C3 |
| U4-theraphotoxin-Pf17 | MRCFSAMFSAEDQLSPQEKGMSDCISLYDSFFYHNLL |  | x | 4’342.8709 | 31.9709 | 37 | 2 | x | 38.85 | 832 | U5-theraphotoxin-Hhn1a | *Haplopelma hainanum* | huwentoxin-1 | 75 | 3.10E-04 | D2Y2C3 |
| U4-theraphotoxin-Pf18 | LCKSDDDCCPHLMCYKYGWCGWDGSVGGSASLLLPNC |  | x | 4’012.6709 | 16.0058 | 37 | 6 | x | 47.28 | 839 | U1-theraphotoxin-Hhn1a | *Haplopelma hainanum* | huwentoxin-1 | 59.3 | 2.80E-06 | P0CH70 |
| U4-theraphotoxin-Pf19 | FSAEDQLSPQERECRYWLGGCEKTSDCCEHLSCSPSVC |  | x | 4’282.7709 | _ | 38 | 6 | x | 52.36 | 5’026 | U5-theraphotoxin-Hhn1a | *Haplopelma hainanum* | huwentoxin-1 | 80 | 3.90E-11 | D2Y2C3 |
| U4-theraphotoxin-Pf20 | ECRYFWGECNDKDLVCCDYLVCKYKWPLSYNICVWNRTFPG | x |  |  |  | 41 | 6 | x |  | 22’399 | U7-theraphotoxin-Hhn1e | *Haplopelma hainanum* | huwentoxin-1 | 77.5 | 2.30E-26 | D2Y2A6 |
| U4-theraphotoxin-Pf21 | NCAKEGELCGWGSRCCHDLYCPAAVVAYCEP | x |  |  |  | 31 | 6 | x |  | 394 | tau-theraphotoxin-Hs1a | *Haplopelma schmidti* | huwentoxin-1 | 73.3 | 1.80E-13 | P0CH43 |
| U4-theraphotoxin-Pf22 | CYASEVEELNLQDEDCNIYSDSCAGSVCRPFSWM |  | x | 3’879.5709 | 16.0071 | 34 | 4 | x | 52.19 | 16’473 | U5-theraphotoxin-Hs1b 2 | *Haplopelma schmidti* | huwentoxin-1 | 31.9 | 4.60E+00 | B3FIS7 |
| U4-theraphotoxin-Pf23 | QEYVPETENCAKEGELCGWGSRCCHDLYCPAALSDM | x | x | 4’006.6209 | -1.0263 | 36 | 5 |  | 47.11 | 1’798 | tau-theraphotoxin-Hs1a | *Haplopelma schmidti* | huwentoxin-1 | 76.5 | 2.10E-20 | P0CH43 |
| U4-theraphotoxin-Pf24 | ESERASACAKEGEVCGWGKRCCDLDNYYCPAGIVPFC |  | x | 4’029.7209 | _ | 37 | 6 | x | 39.19 | 2’676 | tau-theraphotoxin-Hs1a | *Haplopelma schmidti* | huwentoxin-1 | 73.3 | 3.70E-16 | P0CH43 |
| U4-theraphotoxin-Pf25 | ECRYFWGQCGGQEGNCCAHLVCRRKWPNICIWDLTV |  | x | 4’243.9209 | _ | 36 | 6 | x | 55.76 | 7’667 | kappa-theraphotoxin-Hm2a | *Heteroscodra maculata* | huwentoxin-1 | 61.1 | 1.60E-13 | P60993 |
| U4-theraphotoxin-Pf26 | ECRYFWGQCGGQEGNCYFVKLNFSACNCCKSQTLPLH |  | x | 4’233.8209 | _ | 37 | 6 | x | 52.69 | 652 | kappa-theraphotoxin-Hm2a | *Heteroscodra maculata* | huwentoxin-1 | 88.9 | 2.60E-01 | P60993 |
| U4-theraphotoxin-Pf27 | WYLGACKKDSDCCKHLQCHSYWEWCIWDGKVTF | x |  |  |  | 33 | 5 |  |  | 1’627 | tau-theraphotoxin-Pc1c | *Psalmopoeus cambridgei* | huwentoxin-1 | 82.8 | 5.40E-23 | P0C246 |
| U4-theraphotoxin-Pf28 | MCHNKEFMGLCKSDDDCCPHLMCYKYGWCGWDG |  | x | 3’949.4709 | 73.9880 | 33 | 6 | x | 46.26 | 296 | tau-theraphotoxin-Pc1c | *Psalmopoeus cambridgei* | huwentoxin-1 | 55.2 | 1.50E-06 | P0C246 |
| U4-theraphotoxin-Pf29 | CSRYFLGGCTEHSDCCEHLSCKMGLNYCAWDGTF |  | x | 3’878.5382 | 42.0194 | 34 | 6 | x | 61.76 | 10’575 | tau-theraphotoxin-Pc1b | *Psalmopoeus cambridgei* | huwentoxin-1 | 84.4 | 4.30E-20 | P0C245 |
| U4-theraphotoxin-Pf30 | GGCTEHSDCCEHLSCKMESQYKLFTMHCSICPEN |  | x | 3’850.5209 | _ | 34 | 6 | x | 43.39 | 75 | tau-theraphotoxin-Pc1b | *Psalmopoeus cambridgei* | huwentoxin-1 | 82.4 | 2.60E-06 | P0C245 |
| U4-theraphotoxin-Pf31 | ECRWYLGACKKDSDCCKHLQCHSYWEWCIWDGTIS | x | x | 4’252.8209 | _ | 35 | 6 | x | 51.90 | 13’216 | tau-theraphotoxin-Pc1c | *Psalmopoeus cambridgei* | huwentoxin-1 | 81.8 | 3.10E-25 | P0C246 |
| U4-theraphotoxin-Pf32 | AGCRYFLGGCTEHSDCCEHLSCKMGLNYCAWDGTF | x | x | 3’877.5554 | _ | 35 | 6 | x | 52.23 | 8’638 | tau-theraphotoxin-Pc1b | *Psalmopoeus cambridgei* | huwentoxin-1 | 84.8 | 1.60E-21 | P0C245 |
| U4-theraphotoxin-Pf33 | IFECSLSCDIKKEGKACKGKGEKKCGGGWRCKMNFCLKF | x | x | 4’359.11 | _ | 39 | 6 | x | 29.69 | 4’207 | U3-theraphotoxin-Cg1b | *Chilobrachys guangxiensis* | huwentoxin-2 | 84.6 | 2.80E-25 | B1P1A8 |
| U4-theraphotoxin-Pf34 | IFGCTLSCDIKKEGKACKGKGEKKCGGGWRCKFNFCIRF | x |  |  |  | 39 | 6 | x |  | 645 | U3-theraphotoxin-Cg1b | *Chilobrachys guangxiensis* | huwentoxin-2 | 76.9 | 3.10E-22 | B1P1A8 |
| U4-theraphotoxin-Pf35 | CIGEGVPCDENDPRCCSKLECLKPKGYGWWYKSYYCYKKKSS | x | x | 4’914.2209 | _ | 42 | 6 | x | 37.45 | 9’392 | omega-theraphotoxin-Bs2a | *Brachypelma smithi* | huwentoxin-1 | 78 | 1.20E-25 | B3FIV1 |
| U5-theraphotoxin-Pf1 | TAVQESPRACSKQPGESCKDNCDCCGATVVCASVYV |  | x | 3’708.5709 | _ | 36 | 6 | x | 57.54 | 3’910 | toxin-like LSTX-R1 | *Lycosa singoriensis* | HWTX-LSTX | 80.6 | 3.90E-15 | B6DD59 |
| U5-theraphotoxin-Pf2 | ACSKQPGQSCKDNCDCCGATVVCASVYVGGKEKKHCSSKTS | x |  |  |  | 42 | 7 |  |  | 1’266 | toxin-like LSTX-R1 | *Lycosa singoriensis* | HWTX-LSTX | 78 | 4.30E-21 | B6DD59 |
| U5-theraphotoxin-Pf3 | ACSKQPGQSCKDNCDCCGATVVCASVYVGGKEKKHCSSKTSDNGILNVIGQGINLVSNGASVC | x |  |  |  | 63 | 8 | x |  | 9’103 | toxin-like LSTX-R1 | *Lycosa singoriensis* | HWTX-LSTX | 73 | 6.20E-33 | B6DD59 |
| U6-theraphotoxin-Pf1 | TECSRKTWPCETSEDCCDGDCSDTYWTCQLGYGCTRICV | x | x | 4’417.6982 | _ | 39 | 8 | x | 53.05 | 2’211 | U29-theraphotoxin-Cg1a | *Chilobrachys guangxiensis* | insecticidal ABC | 86.5 | 4.20E-27 | B1P1I0 |
| U7-theraphotoxin-Pf1 | CWGANVPCENEKSPCCRGLSCEKTFGYGWWYGSPFCVRKK | x |  |  |  | 41 | 6 | x |  | 7’731 | U26-theraphotoxin-Cg1a | *Chilobrachys guangxiensis* | Jztx-56 | 86.8 | 2.30E-27 | B1P1H6 |
| U7-theraphotoxin-Pf2 | CYIPRRRCLTSQQCCKPYDTVNNFAACGMAWPEDKKRKVNKCYICDNELTLCTR | x |  |  |  | 55 | 8 | x |  | 3’862 | U8-theraphotoxin-Hs1b | *Haplopelma schmidti* | Jztx-72 | 94.4 | 1.30E-42 | B3FIP2 |
| U9-theraphotoxin-Pf1 | SESYRASSCILVGDPCESTCDCCGWTTSCRHSKSAF | x | x | 3’876.5709 | _ | 36 | 6 | x | 49.16 | 16’636 | U32-theraphotoxin-Cg1a | *Chilobrachys guangxiensis* | Tx2 | 67.6 | 2.90E-12 | B1P1I6 |
| U9-theraphotoxin-Pf2 | PDADLIETYMGSESYRASSCILVGDPCESTCDCCGW |  | x | 3’876.5619 | _ | 36 | 5 |  | 50.17 | 3’351 | U32-theraphotoxin-Cg1a | *Chilobrachys guangxiensis* | Tx2 | 63.5 | 9.70E-19 | B1P1I6 |
| U9-theraphotoxin-Pf3 | GSESYRASSCILVGDPCESTCDCCGWTTSCRHSKSARS |  | x | 4’029.6709 | _ | 38 | 6 | x | 40.08 | 352 | U32-theraphotoxin-Cg1a | *Chilobrachys guangxiensis* | Tx2 | 64.1 | 8.30E-14 | B1P1I6 |
| U9-theraphotoxin-Pf4 | YCERYEYPDADLIETYMGSESYRASSCILVGDPCESTCD |  | x | 4’157.7209 | 16.0093 | 39 | 4 | x | 39.58 | 16’636 | U32-theraphotoxin-Cg1a | *Chilobrachys guangxiensis* | Tx2 | 54.1 | 1.70E-07 | B1P1I6 |
| U9-theraphotoxin-Pf5 | ASSCILVGDPCESTCDCCGWTTSCRHSKSAGRIVLQPIQNPA |  | x | 4’393.9709 | _ | 42 | 6 | x | 34.48 | 60 | U32-theraphotoxin-Cg1a | *Chilobrachys guangxiensis* | Tx2 | 75.9 | 3.30E-12 | B1P1I6 |
| U9-theraphotoxin-Pf6 | ASSCILVGDPCESTCDCCGWTTSCRHSKSAGEKVCKEGSKIKGLNTIMKGVAAAKKANCVHKHY | x |  |  |  | 64 | 8 | x |  | 16’636 | U32-theraphotoxin-Cg1a | *Chilobrachys guangxiensis* | Tx2 | 68.9 | 3.60E-27 | B1P1I6 |
