# Supplementary material for: Peptidomic and transcriptomic profiling of four distinct spider venoms
Source: PLoS One. 2017 Mar 17;12(3):e0172966. doi: 10.1371/journal.pone.0172966 (PMC5357004; doi:10.1371/journal.pone.0172966)
Supplement: S5 Table — (DOCX) [file pone.0172966.s005.docx]

| Proposed Name | Predicted/ Detected Mature Sequence | Retrieved by HMM | Detected by MS/MS | Mass [Da] | PTM mass [Da] | Length | Cys number | Complete | Rt [min] | RPKM | Match found in Uniprot | Specie | Family | % Identity | e-value | Uniprot Code |
| --- | --- | --- | --- | --- | --- | --- | --- | --- | --- | --- | --- | --- | --- | --- | --- | --- |
| U1-ctenitoxin-Vf1 | KENCGTTGHDCEDVKCCEGYRCLYTARTGRLWMCL |  | x | 4’014.7209 | _ | 35 | 6 | x | 44.62 | 2’033 | U6-agatoxin-Ao1a | *Agelena orientalis* | _ | 48 | 7.50E-01 | Q5Y4W0 |
| U1-ctenitoxin-Vf2 | GYCAEKGVKCHNIHCCENLRCKCNDDRSSCVCRKNKVS | x |  |  |  | 38 | 8 | x |  | 1’734 | U4-agatoxin-Ao1a | *Agelena orientalis* | _ | 76.5 | 1.70E-16 | Q5Y4U5 |
| U1-ctenitoxin-Vf3 | FCVKTGDSCSGLCQCCDPEADCECRGEFIPGKSPCSCKDRGNVKVCKWKQEMCRINRPKNC | x |  |  |  | 61 | 12 | x |  | 634 | U9-agatoxin-Ao1a | *Agelena orientalis* | _ | 39.6 | 4.10E-05 | Q5Y4U3 |
| U1-ctenitoxin-Vf4 | NECCSQDNCRFGYMCCAEPCGNVCRVKVDTALGIPVEEDSDCKLGEVDKKWYQKALSVFSRIEESEETPNASMQ | x |  |  |  | 74 | 8 | x |  | 1’413 | U7-agatoxin-Ao1a | *Agelena orientalis* | _ | 71.9 | 4.40E-31 | Q5Y4V9 |
| U2-ctenitoxin-Vf1 | STKCTKRNHDCTGDRHRCCRGKMFKDVCMCFYKEGNETAR | x |  |  |  | 40 | 6 | x |  | 53 | CSTX-14 | *Cupiennius salei* | CSTX | 82.8 | 1.20E-17 | B3EWS7 |
| U2-ctenitoxin-Vf2 | SCFTDADCESDECCISMVFVRGFCHTLGKEGDHCIDKPADTEHGKKNLFGCPCREGYKCIAEVTQEGEKTVFKNPTCREGGE | x |  |  |  | 82 | 10 | x |  | 192 | CSTX-20 | *Cupiennius salei* | _ | 32.9 | 5.10E-07 | B3EWT5 |
| U3-ctenitoxin-Vf1 | EKICADKGDRCDKGLKCCKGPCVVSSLSFNIMISKCIR | x |  |  |  | 38 | 6 | x |  | 2’017 | purotoxin-1 | *Geolycosa sp* | _ | 57.9 | 4.60E+00 | P86269 |
| U4-ctenitoxin-Vf1 | AGQSCGETECGKGECCAGSFYHRNCRPLSDNGQPCESPNESDNYSTACPCKDGLVCNPIRRCQRS | x |  |  |  | 65 | 10 | x |  | 849 | U19-ctenitoxin-Pn1a | *Phoneutria nigriventer* | _ | 52.4 | 2.00E-25 | P83997 |
| U4-ctenitoxin-Vf2 | VQGKYCISTAECGEGMCCTGGSFNKHCQSLSDEGRPCQRPNEYEDYRTGCPCKEGLFCSVINYCQKA | x |  |  |  | 67 | 10 | x |  | 1’473 | U19-ctenitoxin-Pn1a | *Phoneutria nigriventer* | _ | 69.7 | 2.50E-39 | P83997 |
| U5-ctenitoxin-Vf1 | EKCRKTCDCCERPNAICECTAEFWVGKSYCYCKEGELINCQLKKQGCPRNQASK | x |  |  |  | 54 | 10 | x |  | 4’518 | U13-lycotoxin-Ls1b | *Lycosa singoriensis* | spider agouti | 34.5 | 3.90E-04 | B6DD28 |
| U6-ctenitoxin-Vf1 | TKTCSLEYKINDCCKQADCPAGSTCCKLPCGNSCQRESPVATN | x |  |  |  | 43 | 8 | x |  | 51 | U20-lycotoxin-Ls1d | *Lycosa singoriensis* | spider wap-2 | 62.9 | 5.50E-10 | B6DCY1 |
| U6-ctenitoxin-Vf2 | TKTCSLDYKINDCCKQADCPAGSTCCKLPCGNSCQRESPVATNGVPVKD | x |  |  |  | 49 | 8 | x |  | 49 | U20-lycotoxin-Ls1d | *Lycosa singoriensis* | spider wap-2 | 62.9 | 6.90E-10 | B6DCY1 |
| U6-ctenitoxin-Vf3 | EDPGNCPSFTNKECPFNYKINDCCAQADCPAGAICCVQPCGNVCRRKSETPKGTP | x |  |  |  | 55 | 9 |  |  | 890 | U20-lycotoxin-Ls1c | *Lycosa singoriensis* | spider wap-2 | 58 | 1.10E-19 | B6DD62 |
| U6-ctenitoxin-Vf4 | RKTCSLSYKTHDCCKQSDCPPGNTCCELPCGNACQRESPVATNGVPVRDGAKPCK | x |  |  |  | 55 | 9 |  |  | 1’514 | U20-lycotoxin-Ls1d | *Lycosa singoriensis* | spider wap-2 | 60 | 1.00E-10 | B6DCY1 |
| U6-ctenitoxin-Vf5 | VASIVQRQRTKTCSLDYKINDCCKQADCPAGSTCCKLPCGNSCQRESPVATNGVPVK | x |  |  |  | 57 | 8 | x |  | 64 | U20-lycotoxin-Ls1d | *Lycosa singoriensis* | spider wap-2 | 56.1 | 2.60E-12 | B6DCY1 |
| U6-ctenitoxin-Vf6 | TKTCSLDYKINDCCKQADCPAGSTCCKLPCGNSCQRESPVATNGVPVKDGEYCVEGTDDGY | x |  |  |  | 61 | 9 |  |  | 1’629 | U20-lycotoxin-Ls1d | *Lycosa singoriensis* | spider wap-2 | 50.9 | 2.20E-12 | B6DCY1 |
| U6-ctenitoxin-Vf7 | TKTCSLDYKINDCCKQADCPAGSTCCKLPCGNSCQRESPVATNGVPVKDGEYCVEGTETDIK | x |  |  |  | 62 | 9 |  |  | 30 | U20-lycotoxin-Ls1d | *Lycosa singoriensis* | spider wap-2 | 50.9 | 2.40E-12 | B6DCY1 |
| U6-ctenitoxin-Vf8 | GGKYCPEPKRKSCPLDYKINDCCKQSDCPAGSTCCKLPCGNVCQRESPVATNGVPVKDGEPCVEGHDDGY | x |  |  |  | 70 | 10 | x |  | 12’472 | U20-lycotoxin-Ls1c | *Lycosa singoriensis* | spider wap-2 | 50.7 | 1.50E-18 | B6DD62 |
| U6-ctenitoxin-Vf9 | GGKYCPEPKKPTCMLDYKINECCKESDCSAGSICCKLPCGNACQRESPFATNGVPVKDGEHCVRGIDVRY | x |  |  |  | 70 | 10 | x |  | 894 | U20-lycotoxin-Ls1d | *Lycosa singoriensis* | spider wap-2 | 47 | 5.20E-17 | B6DCY1 |
| U7-ctenitoxin-Vf1 | EDKLGRCTLGDCDPSKSHCQCCGDFTYCGCFWDISWLGNCRCRPGTRVSCFEKR | x |  |  |  | 54 | 10 | x |  | 2’476 | mu-ctenitoxin-Pn1a | *Phoneutria nigriventer* | Tx1 | 51.5 | 9.00E-07 | P17727 |
| U7-ctenitoxin-Vf2 | CRKTCDCCERPNAICECTAEFWVGKSYCYCKER | x |  |  |  | 33 | 8 | x |  | 10 | delta-ctenitoxin-Pr2d | *Phoneutria reidyi* | Tx2 | 44.8 | 1.40E+00 | P83904 |
| U7-ctenitoxin-Vf3 | GCAKRGKSCDHVKCCPNSACRCNGFRTNCRCGSPGLFTMIG | x |  |  |  | 41 | 8 | x |  | 2’669 | omega-agatoxin-Aa5a | *Agelenopsis aperta* | Tx3 | 60 | 7.10E-04 | P0DL48 |
| U7-ctenitoxin-Vf4 | CIDVYETCNKGIPCCEDRPCKCNIVMDNCICKKTIAELFR | x |  |  |  | 40 | 8 | x |  | 8 | kappa-ctenitoxin-Pn1a | *Phoneutria nigriventer* | Tx3 | 72.5 | 2.30E-18 | O76200 |
| U7-ctenitoxin-Vf5 | APKCIDVYETCNKGIPCCEDRPCRCNIVMDNCICKKTIAELFGGK | x |  |  |  | 45 | 8 | x |  | 9’601 | kappa-ctenitoxin-Pn1a | *Phoneutria nigriventer* | Tx3 | 70.5 | 1.20E-18 | O76200 |
| U7-ctenitoxin-Vf6 | CYETCCEDRPCRCNIVMDNCICKKTIAELFGGNNYYTRQDEVTCLL | x |  |  |  | 46 | 8 | x |  | 24 | kappa-ctenitoxin-Pn1a | *Phoneutria nigriventer* | Tx3 | 76.7 | 4.10E-15 | O76200 |
| U7-ctenitoxin-Vf7 | APKCIDVYETCNKGIPCCEDRPCKCNIVMDNCICKNSKDVSSKNCSAENNFYTRQDE | x |  |  |  | 57 | 9 |  |  | 684 | kappa-ctenitoxin-Pn1a | *Phoneutria nigriventer* | Tx3 | 70.6 | 2.60E-13 | O76200 |
| U7-ctenitoxin-Vf8 | APKCIDVYETCNKGNTLAVKTDLANANIVMDNCICKKTIAELFGGNNFYTRQDEQYWHRLVNIHNHLKLK | x |  |  |  | 70 | 4 | x |  | 72 | kappa-ctenitoxin-Pn1a | *Phoneutria nigriventer* | Tx3 | 52.4 | 3.60E-08 | O76200 |
| U7-ctenitoxin-Vf9 | APKCIDVYETCNKGIPCCEDRPCRCNIVMDNCICKKTIAELFGGSYLPSLNSVQLDLKEKSLQIIIMSSKVFLFILLLLSLQFLEQML | x |  |  |  | 88 | 8 | x |  | 48 | kappa-ctenitoxin-Pn1a | *Phoneutria nigriventer* | Tx3 | 69 | 5.00E-15 | O76200 |
| U8-ctenitoxin-Vf1 | YKKCNQTGKDCSNDCDCCNKWTCKCPIWGLFGCSCVFGDSMV | x |  |  |  | 42 | 9 |  |  | 604 | U18-ctenitoxin-Pn1a | *Phoneutria nigriventer* | type II/III omega-agatoxin | 77.5 | 8.90E-22 | P83999 |
| U8-ctenitoxin-Vf2 | YKKCNQTGKDCSNDCDCCNKWTYCKCPIWGLFGCSCVFGDSMVCVRKREQCKDPAVMDFQKEAAFLKEEECT | x |  |  |  | 72 | 12 | x |  | 2’552 | U18-ctenitoxin-Pn1a | *Phoneutria nigriventer* | type II/III omega-agatoxin | 79.5 | 4.20E-26 | P83999 |
| U8-ctenitoxin-Vf3 | SCKKREEACVTDCDCCGGFYICHYPAVIKYLGVGGSCIYGNKYDCER | x |  |  |  | 47 | 8 | x |  | 5’467 | U7-ctenitoxin-Pr1a | *Phoneutria reidyi* | type II/III omega-agatoxin | 49 | 3.30E-11 | P84031 |
| U8-ctenitoxin-Vf4 | SCKKRAEACKTDCDCCGGFYKCHFPAGLKYFGFGGSCVYGNKYDCER | x |  |  |  | 47 | 8 | x |  | 481 | U7-ctenitoxin-Pr1a | *Phoneutria reidyi* | type II/III omega-agatoxin | 56.9 | 7.70E-15 | P84031 |
| U9-ctenitoxin-Vf1 | CASQGEECADGDDCSCCGDRGKCDCNWPNKPGCFCMQGMAYTAMKKAMICGW | x |  |  |  | 52 | 10 | x |  | 6’030 | U12-lycotoxin-Ls1a | *Lycosa singoriensis* | U12-lycotoxin | 54.3 | 4.10E-11 | B6DD17 |
| U9-ctenitoxin-Vf2 | CASQGEECADGDDCSCCGDRGKCDCNWPNKPGCFCMQGMAYTAMKKADDYLIKTFNF | x |  |  |  | 57 | 9 |  |  | 826 | U12-lycotoxin-Ls1a | *Lycosa singoriensis* | U12-lycotoxin | 54.3 | 4.40E-11 | B6DD17 |
| U9-ctenitoxin-Vf3 | CIPKHYECTHHKDECCKGHLFHYKCRCYKIAGDKGEESKR | x |  |  |  | 40 | 6 | x |  | 131 | U1-lycotoxin-Ls1hh | *Lycosa singoriensis* | U1-lycotoxin | 60 | 3.50E-15 | B6DCN9 |
| U9-ctenitoxin-Vf4 | QCIRYEHVCSFNKGECCTGLKCECYDRYIKGEKGEEKCWCIEKDVMYKKRGE | x |  |  |  | 52 | 8 | x |  | 235 | U6-lycotoxin-Ls1g | *Lycosa singoriensis* | U6-lycotoxin | 53.2 | 5.90E-14 | B6DCV8 |
| U9-ctenitoxin-Vf5 | QCIQYEHVCSFNIGKCCPGLKCECYDRYIKGEKGEEKCWCIEKDVMYKKRGE | x |  |  |  | 52 | 8 | x |  | 236 | U6-lycotoxin-Ls1g | *Lycosa singoriensis* | U6-lycotoxin | 53.2 | 2.50E-13 | B6DCV8 |
| venom kunitz type-like peptide Vf1 | IMNAASEACDLPAETGVCRGFFPRYYFDRTTGQCQKFVYGGCGGNENNFETVEECQRTCA | x |  |  |  | 60 | 6 | x |  | 119 | kunitz-type serine protease inhibitor Bt-KTI | *Bombus terrestris* | venom kunitz-type | 53.8 | 5.40E-20 | D8KY58 |
| venom kunitz type-like peptide Vf2 | CSLHSETGPCKAMMPRFYFNGNKCESFIFGGCKGNKNNFLSVEECEQKCGPKTSEE | x |  |  |  | 56 | 6 | x |  | 947 | kunitz-type U19-barytoxin-Tl1a | *Trittame loki* | venom kunitz-type | 57.1 | 2.90E-19 | W4VSH9 |
