# Supplementary material for: Peptidomic and transcriptomic profiling of four distinct spider venoms
Source: PLoS One. 2017 Mar 17;12(3):e0172966. doi: 10.1371/journal.pone.0172966 (PMC5357004; doi:10.1371/journal.pone.0172966)
Supplement: S6 Table — (DOCX) [file pone.0172966.s006.docx]

| Proposed Name | Predicted/ Detected Mature Sequence | Retrieved by HMM | Detected by MS/MS | Mass [Da] | PTM mass [Da] | Length | Cys number | Complete | Rt [min] | RPKM | Match found in Uniprot | Specie | Family | % Identity | e-value | Uniprot Code |
| --- | --- | --- | --- | --- | --- | --- | --- | --- | --- | --- | --- | --- | --- | --- | --- | --- |
| U1-theritoxin-Lm1 | KQCIKKHYECTHDKRNCCVGKVFQYTCKCYDYTNSAGVVEPRCKCTKSILGALTDFGVNLWNRVTG | x |  |  |  | 66 | 8 | x |  | 1’688 | U1-ctenitoxin-Cs1a | *Cupiennius salei* | CSTX | 44.0 | 1.70E-12 | P58604 |
| U2-theritoxin-Lm1 | GCTDISQAEFDEKNANCIKCGEKDFGEEIVKRCRDKCFTDNFYQSCVDLLNDV | x |  |  |  | 54 | 6 | x |  | 2’210 | alpha-latrotoxin associated low molecular weight | *Latrodectus hesperus* | _ | 88.7 | 1.10E-34 | V9QFH5 |
| U2-theritoxin-Lm2 | GCTDISQAEFDEKNANCIKCGEKDFGEEIVKRCRDKCFTDNFYQSCVDLLNDVYEEK | x |  |  |  | 57 | 6 | x |  | 2’265 | alpha-latrotoxin associated low molecular weight | *Latrodectus hesperus* | _ | 89.5 | 1.60E-38 | V9QFH5 |
| U2-theritoxin-Lm3 | CEDLHKEGVVFSLCKTNCFTTEYFTNCVKDLEEAEKEPP | x |  |  |  | 40 | 4 | x |  | 2’623 | alpha-latrotoxin associated low molecular weight 2 | *Latrodectus hesperus* | arthropod CHH/MIH/GIH/VIH hormone | 97.4 | 8.40E-29 | V9QEI7 |
| U2-theritoxin-Lm4 | LKCEDLHKEGVVFSLCKTNCFTTEYFTNCVKDLEEAEKEPPE | x |  |  |  | 42 | 4 | x |  | 4’232 | alpha-latrotoxin associated low molecular weight 2 | *Latrodectus hesperus* | arthropod CHH/MIH/GIH/VIH hormone | 95.2 | 2.20E-30 | V9QEI7 |
| peptidase-like peptide Lm1 | LQIMDTPCCSNIYNMEGYSYVDESVLCAYRDGVDTCAFDSGGPLMKK | x |  |  |  | 47 | 4 | x |  | 1’078 | venom protease | *Bombus pensylvanicus* | peptidase S1 | 56.7 | 3.70E-02 | Q7M4I3 |
| U3-theritoxin-Lm1 | DECTPLTHDCTHDRHSCCRGPTFKYKCDCLYPFDNSTSAWDQTELCFCVEPGVHHFLDEVMDKTIGIFG | x |  |  |  | 69 | 8 | x |  | 2’214 | purotoxin-2 | *Geolycosa sp.* | spider toxin CSTX | 54.1 | 2.50E-22 | B3EWH0 |
| U3-theritoxin-Lm2 | SECTPLTHDCTDDRHNCCRGPTFKYKCECLHPYINETNTWDEKELCFCVEPGVHHWFDEAVDKAGSFFW | x |  |  |  | 69 | 8 | x |  | 189 | purotoxin-2 | *Geolycosa sp.* | spider toxin CSTX | 46.8 | 1.00E-16 | B3EWH0 |
| U3-theritoxin-Lm3 | DECTPLTHDCTHDRHSCCRGPTFEYRCECLYPFDESTGTWDTKEMCFCIEPGLHHFFDQHLEKA | x |  |  |  | 64 | 8 | x |  | 672 | purotoxin-2 | *Geolycosa sp.* | spider toxin CSTX | 51.6 | 2.00E-19 | B3EWH0 |
| U3-theritoxin-Lm4 | SNECTPLTHDCTDDRHNCCRGPTFKYKCECLHPYINETNTWDEKELCFCVEPGVHHWFDEAVDKAGSFLG | x |  |  |  | 70 | 8 | x |  | 654 | purotoxin-2 | *Geolycosa sp.* | spider toxin CSTX | 45.5 | 7.60E-17 | B3EWH0 |
