# Supplementary material for: Peptidomic and transcriptomic profiling of four distinct spider venoms
Source: PLoS One. 2017 Mar 17;12(3):e0172966. doi: 10.1371/journal.pone.0172966 (PMC5357004; doi:10.1371/journal.pone.0172966)
Supplement: S7 Table — (DOCX) [file pone.0172966.s007.docx]

| Peptide name | Accession number GenBank |
| --- | --- |
| U4-theraphotoxin-Pf32 | KY689243 |
| U4-theraphotoxin-Pf31 | KY689244 |
| U4-theraphotoxin-Pf7 | KY689245 |
| U1-theraphotoxin-Pf7 | KY689246 |
| U8-ctenitoxin-Vf4 | KY689247 |
| U7-ctenitoxin-Vf4 | KY689248 |
| CRISP-like peptide Hdb1 | KY689249 |
| U2-ctenitoxin-Vf2 | KY689250 |
| U6-sparatoxin-Hdb22 | KY689251 |
| U4-ctenitoxin-Vf2 | KY689252 |
| U4-theraphotoxin-Pf17 | KY689253 |
| U4-sparatoxin-Hdb6 | KY689254 |
| U7-ctenitoxin-Vf7 | KY689255 |
| U6-sparatoxin-Hdb23 | KY689256 |
| U4-theraphotoxin-Pf25 | KY689257 |
| U5-theraphotoxin-Pf3 | KY689258 |
| U4-theraphotoxin-Pf33 | KY689259 |
| U1-sparatoxin-Hdb3 | KY689260 |
| U9-ctenitoxin-Vf2 | KY689261 |
| U4-theraphotoxin-Pf22 | KY689262 |
| peptidase-like peptide Lm1 | KY689263 |
| U5-theraphotoxin-Pf1 | KY689264 |
| U1-ctenitoxin-Vf4 | KY689265 |
| U7-theraphotoxin-Pf1 | KY689266 |
| U2-theritoxin-Lm2 | KY689267 |
| U9-sparatoxin-Hdb2 | KY689268 |
| U6-sparatoxin-Hdb17 | KY689269 |
| U8-sparatoxin-Hdb2 | KY689270 |
| U2-theraphotoxin-Pf3 | KY689271 |
| U4-sparatoxin-Hdb13 | KY689272 |
| CRISP-like peptide Hdb4 | KY689273 |
| U1-sparatoxin-Hdb2 | KY689274 |
| U4-theraphotoxin-Pf18 | KY689275 |
| U7-ctenitoxin-Vf1 | KY689276 |
| U1-theraphotoxin-Pf2 | KY689277 |
| U8-ctenitoxin-Vf2 | KY689278 |
| U3-theritoxin-Lm3 | KY689279 |
| U4-theraphotoxin-Pf10 | KY689280 |
| U6-sparatoxin-Hdb18 | KY689281 |
| U7-ctenitoxin-Vf3 | KY689282 |
| U5-ctenitoxin-Vf1 | KY689283 |
| U4-theraphotoxin-Pf14 | KY689284 |
| U10-sparatoxin-Hdb1 | KY689285 |
| U7-ctenitoxin-Vf6 | KY689286 |
| U6-theraphotoxin-Pf1 | KY689287 |
| CRISP-like peptide Pf1 | KY689288 |
| venom kunitz type-like peptide Vf2 | KY689289 |
| U4-sparatoxin-Hdb17 | KY689290 |
| U10-theraphotoxin-Pf1 | KY689291 |
| U4-theraphotoxin-Pf15 | KY689292 |
| U6-ctenitoxin-Vf5 | KY689293 |
| U8-sparatoxin-Hdb1 | KY689294 |
| U7-sparatoxin-Hdb3 | KY689295 |
| U4-theraphotoxin-Pf16 | KY689296 |
| U1-theraphotoxin-Pf1 | KY689297 |
| U9-ctenitoxin-Vf5 | KY689298 |
| U6-sparatoxin-Hdb19 | KY689299 |
| U6-sparatoxin-Hdb21 | KY689300 |
| U7-sparatoxin-Hdb6 | KY689301 |
| U7-sparatoxin-Hdb4 | KY689302 |
| U9-sparatoxin-Hdb1 | KY689303 |
| U7-ctenitoxin-Vf2 | KY689304 |
| U6-ctenitoxin-Vf9 | KY689305 |
| U6-sparatoxin-Hdb13 | KY689306 |
| U1-theraphotoxin-Pf6 | KY689307 |
| U7-ctenitoxin-Vf8 | KY689308 |
| U9-theraphotoxin-Pf3 | KY689309 |
| U3-sparatoxin-Hdb1 | KY689310 |
| U4-theraphotoxin-Pf29 | KY689311 |
| U4-sparatoxin-Hdb14 | KY689312 |
| U4-theraphotoxin-Pf12 | KY689313 |
| U6-sparatoxin-Hdb11 | KY689314 |
| U6-ctenitoxin-Vf2 | KY689315 |
| U1-theraphotoxin-Pf3 | KY689316 |
| U4-theraphotoxin-Pf35 | KY689317 |
| U2-theritoxin-Lm3 | KY689318 |
| U4-theraphotoxin-Pf5 | KY689319 |
| U1-theritoxin-Lm1 | KY689320 |
| U2-theraphotoxin-Pf1 | KY689321 |
| U2-theraphotoxin-Pf2 | KY689322 |
| U3-theritoxin-Lm4 | KY689323 |
| U4-theraphotoxin-Pf27 | KY689324 |
| U6-ctenitoxin-Vf7 | KY689325 |
| U4-theraphotoxin-Pf2 | KY689326 |
| CRISP-like peptide Pf3 | KY689327 |
| U4-theraphotoxin-Pf21 | KY689328 |
| U9-theraphotoxin-Pf6 | KY689329 |
| U8-ctenitoxin-Vf3 | KY689330 |
| CRISP-like peptide Pf2 | KY689331 |
| U5-theraphotoxin-Pf2 | KY689332 |
| U4-theraphotoxin-Pf26 | KY689333 |
| U8-sparatoxin-Hdb3 | KY689334 |
| U1-theraphotoxin-Pf4 | KY689335 |
| U6-sparatoxin-Hdb20 | KY689336 |
| U7-sparatoxin-Hdb2 | KY689337 |
| U4-theraphotoxin-Pf6 | KY689338 |
| U1-theraphotoxin-Pf5 | KY689339 |
| U9-theraphotoxin-Pf2 | KY689340 |
| U4-ctenitoxin-Vf1 | KY689341 |
| U4-theraphotoxin-Pf34 | KY689342 |
| U2-theritoxin-Lm4 | KY689343 |
| U8-ctenitoxin-Vf1 | KY689344 |
| CRISP-like peptide Hdb3 | KY689345 |
| U9-theraphotoxin-Pf4 | KY689346 |
| PLA2-like peptide Hdb1 | KY689347 |
| U4-theraphotoxin-Pf13 | KY689348 |
| U4-theraphotoxin-Pf11 | KY689349 |
| U1-ctenitoxin-Vf3 | KY689350 |
| U4-theraphotoxin-Pf4 | KY689351 |
| U4-sparatoxin-Hdb16 | KY689352 |
| U6-sparatoxin-Hdb15 | KY689353 |
| U4-theraphotoxin-Pf24 | KY689354 |
| U4-theraphotoxin-Pf23 | KY689355 |
| U7-sparatoxin-Hdb1 | KY689356 |
| U4-sparatoxin-Hdb15 | KY689357 |
| U4-theraphotoxin-Pf3 | KY689358 |
| U1-ctenitoxin-Vf1 | KY689359 |
| U4-theraphotoxin-Pf28 | KY689360 |
| U2-ctenitoxin-Vf1 | KY689361 |
| U4-theraphotoxin-Pf1 | KY689362 |
| U7-theraphotoxin-Pf2 | KY689363 |
| U9-sparatoxin-Hdb4 | KY689364 |
| U6-ctenitoxin-Vf1 | KY689365 |
| U7-sparatoxin-Hdb5 | KY689366 |
| U4-theraphotoxin-Pf9 | KY689367 |
| U4-theraphotoxin-Pf20 | KY689368 |
| venom kunitz type-like peptide Vf1 | KY689369 |
| U5-sparatoxin-Hdb1 | KY689370 |
| U4-theraphotoxin-Pf19 | KY689371 |
| U6-sparatoxin-Hdb14 | KY689372 |
| U6-sparatoxin-Hdb16 | KY689373 |
| U9-ctenitoxin-Vf4 | KY689374 |
| U6-sparatoxin-Hdb12 | KY689375 |
| U3-theraphotoxin-Pf1 | KY689376 |
| U9-ctenitoxin-Vf1 | KY689377 |
| U6-ctenitoxin-Vf8 | KY689378 |
| U9-sparatoxin-Hdb3 | KY689379 |
| U9-ctenitoxin-Vf3 | KY689380 |
| U4-theraphotoxin-Pf30 | KY689381 |
| U6-ctenitoxin-Vf3 | KY689382 |
| U3-ctenitoxin-Vf1 | KY689383 |
| U1-ctenitoxin-Vf2 | KY689384 |
| U2-theritoxin-Lm1 | KY689385 |
| U3-theritoxin-Lm2 | KY689386 |
| CRISP-like peptide Hdb2 | KY689387 |
| U7-ctenitoxin-Vf9 | KY689388 |
| U6-ctenitoxin-Vf4 | KY689389 |
| U6-ctenitoxin-Vf6 | KY689390 |
| U9-theraphotoxin-Pf1 | KY689391 |
| U3-theritoxin-Lm1 | KY689392 |
| U4-sparatoxin-Hdb2 | KY689393 |
| U7-ctenitoxin-Vf5 | KY689394 |
| U4-theraphotoxin-Pf8 | KY689395 |
| U2-sparatoxin-Hdb1 | KY689396 |
| U9-theraphotoxin-Pf5 | KY689397 |
