# Supplementary figures and images for: Peptidomic and transcriptomic profiling of four distinct spider venoms
Source: PLoS One. 2017 Mar 17;12(3):e0172966. doi: 10.1371/journal.pone.0172966 (PMC5357004; doi:10.1371/journal.pone.0172966)

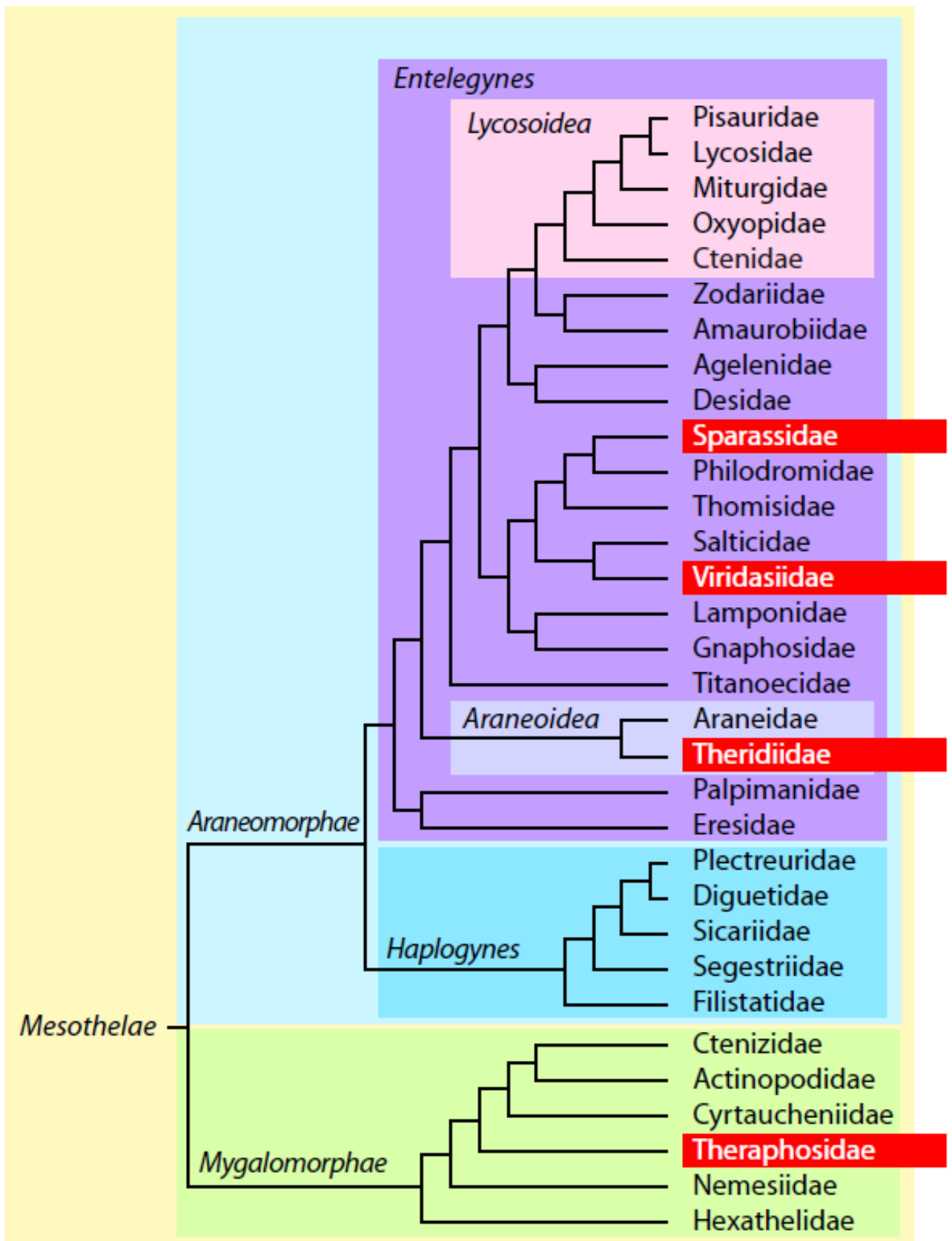

Supplement: S1 Fig — These represent 32 families out of 114. Modified from Kuhn-Nentwig et al. [1] with adaptations following Polotow et al. [2]. (PDF) [file pone.0172966.s008.pdf]

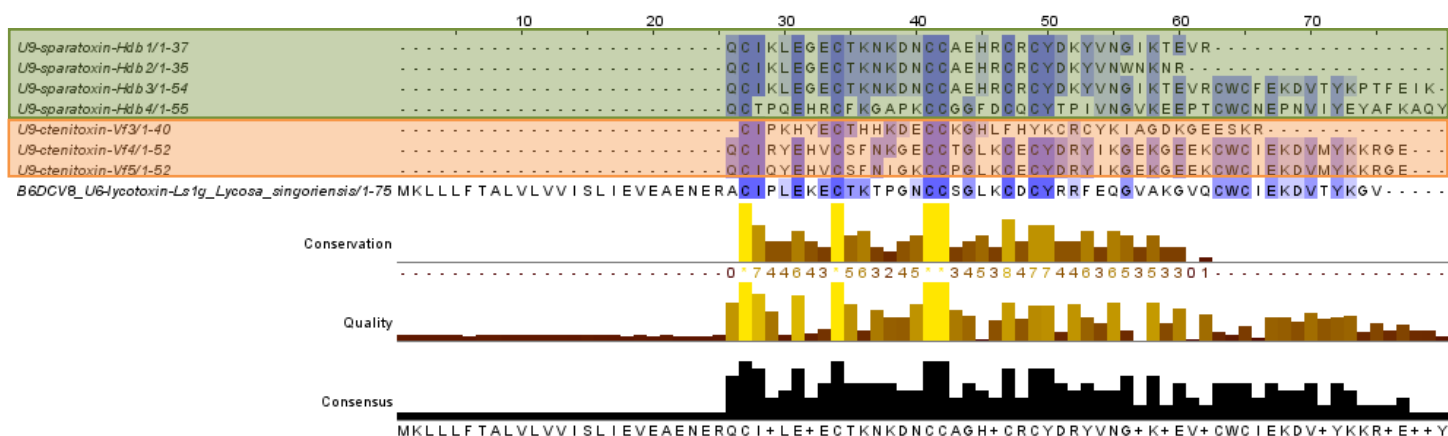

Supplement: S3 Fig — The last reference sequence is the U6-lycotoxin-Ls1g from Lycosa singoriensis (Lycosidae) (Uniprot, accession number B6DCV8). (PDF) [file pone.0172966.s010.pdf]

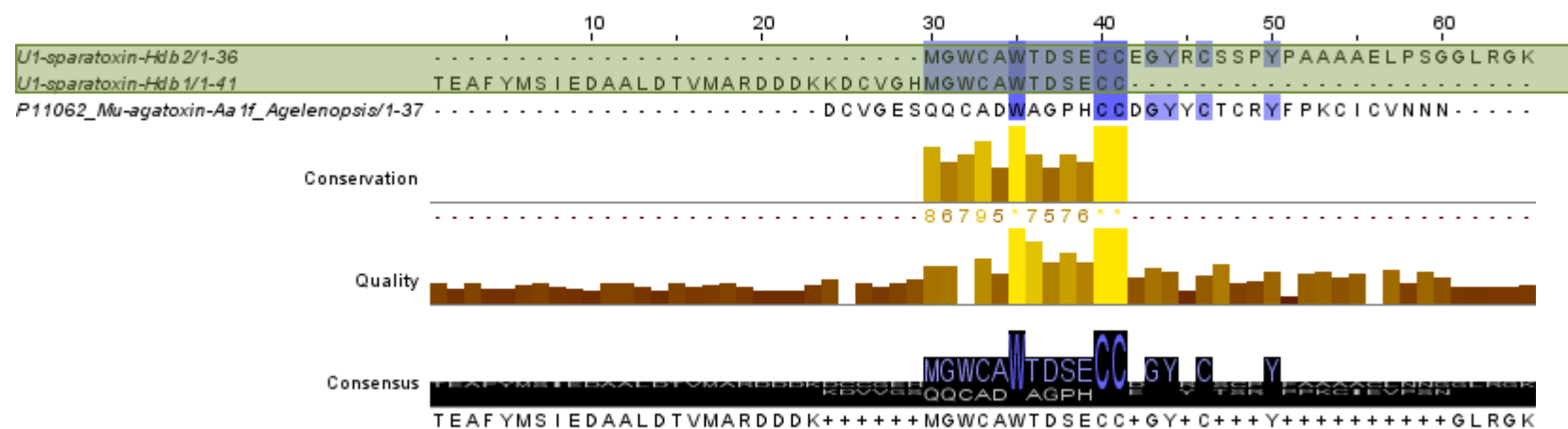

Supplement: S4 Fig — The last reference sequence is the Mu-agatoxin-Aa1f from Agelenopsis aperta (Agelenidae) (Uniprot, accession number P11062). (PDF) [file pone.0172966.s011.pdf]

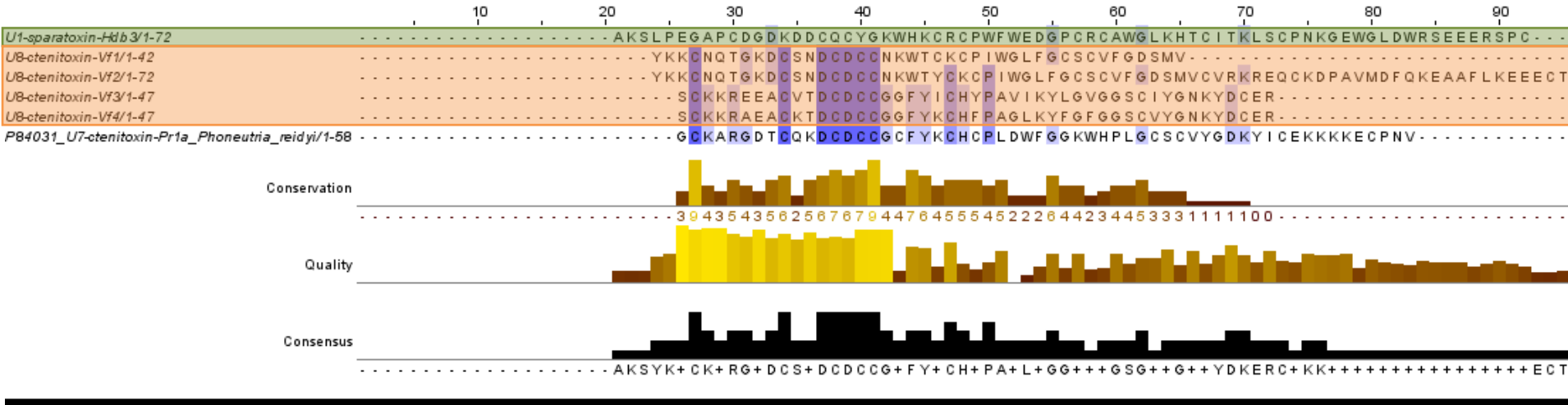

Supplement: S5 Fig — The last reference sequence is the U7-ctenitoxin-Pr1a from Phoneutria reidyi (Ctenidae) (Uniprot, accession number P84031). (PDF) [file pone.0172966.s012.pdf]

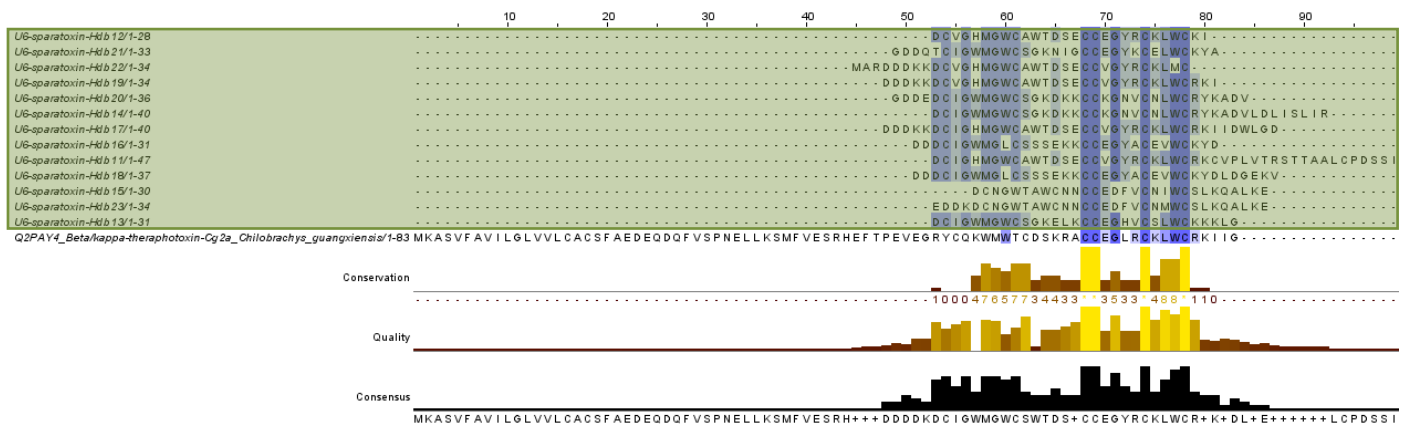

Supplement: S6 Fig — The last reference sequence is the beta/kappa-theraphotoxin-Cg2a from Chilobrachys guangxiensis (Theraphosidae) (Uniprot, accession number Q2PAY4). (PDF) [file pone.0172966.s013.pdf]
