# Supplementary material for: Peptidomic and transcriptomic profiling of four distinct spider venoms
Source: PLoS One. 2017 Mar 17;12(3):e0172966. doi: 10.1371/journal.pone.0172966 (PMC5357004; doi:10.1371/journal.pone.0172966)
Supplement: S2 Fig — The last reference sequence is the Omega-sparatoxin-Hv1b from Heteropoda venatoria (Sparassidae) (Uniprot, accession number P61790). (PDF) [file pone.0172966.s009.pdf]

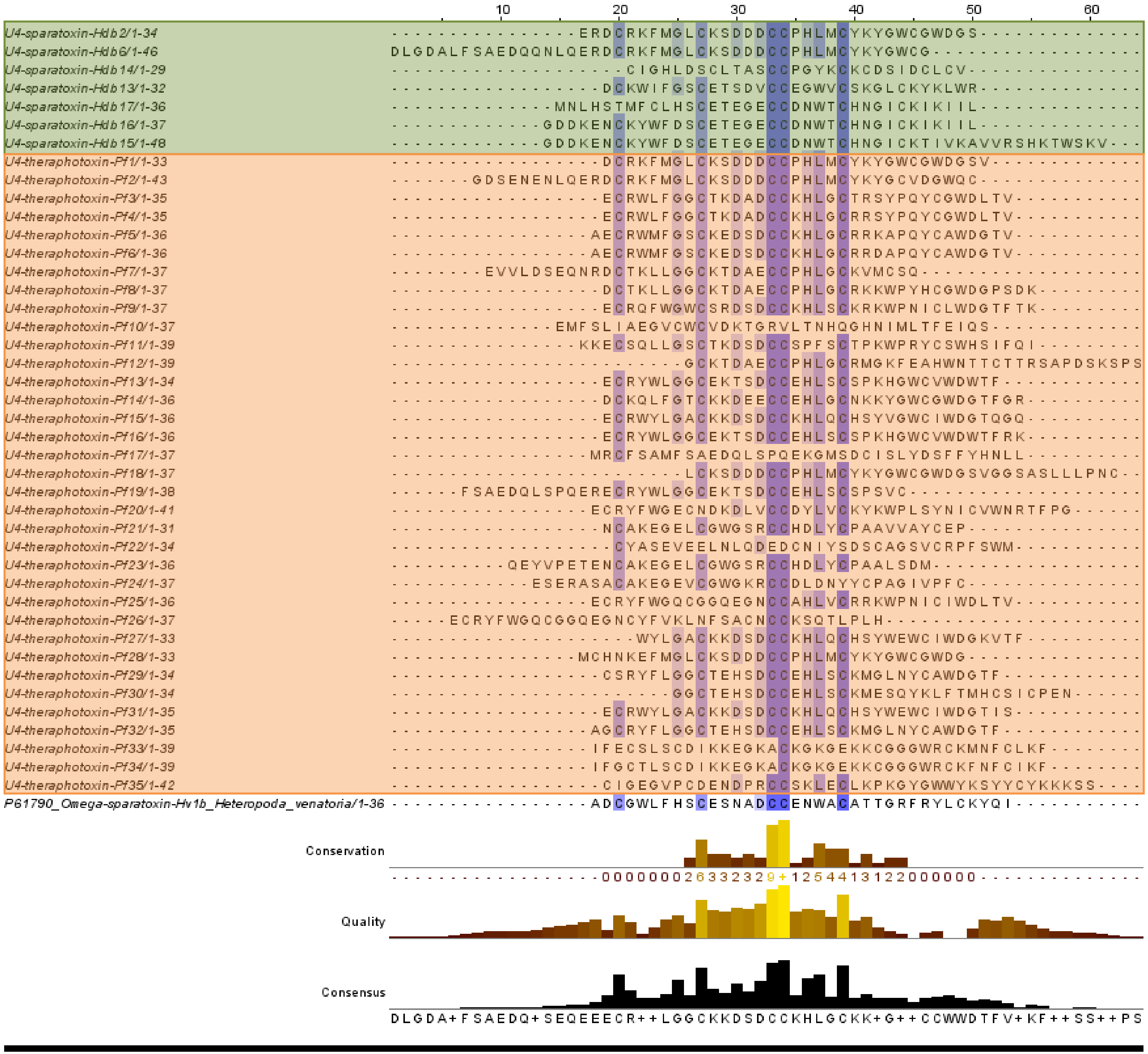

10 20 30 40 50 60
U4-sparatoxin-Hdb2/1-34 ERDCRKFMGLCKSDDDCCPHLMCYKYGWCGWDGS
U4-sparatoxin-Hdb6/1-46 DLGDALFSAEDQQNLQERDCRKFMGLCKSDDDCCPHLMCYKYGWCG
U4-sparatoxin-Hdb14/1-29 CIGHLDSCLTASCCPGYKCKCDSIDCLCV
U4-sparatoxin-Hdb13/1-32 DCKWIFGSCETSDVCCEGWVCSKGLCKYKLWR
U4-sparatoxin-Hdb17/1-36 MNLHSTMFCLHSCETEGECCDNWTCHNGICKIKIIL
U4-sparatoxin-Hdb16/1-37 GDDKENCKYWFDSCETEGECCDNWTCHNGICKIKIIL
U4-sparatoxin-Hdb15/1-48 GDDKENCKYWFDSCETEGECCDNWTCHNGICKTIVKAVVRSHKTWSKV
U4-theraphotoxin-Pf1/1-33 DCRKFMGLCKSDDDCCPHLMCYKYGWCGWDGSV
U4-theraphotoxin-Pf2/1-43 GDSENENLQERDCRKFMGLCKSDDDCCPHLMCYKYGCVDGWQC
U4-theraphotoxin-Pf3/1-35 ECRWLFGGCTKDADCCKHLGCTRSYPQYCGWDLTV
U4-theraphotoxin-Pf4/1-35 ECRWLFGGCTKDADCCKHLGCRRSYPQYCGWDLTV
U4-theraphotoxin-Pf5/1-36 AECRWMFGSCKEDSDCCKHLGCRRKAPQYCAWDGTV
U4-theraphotoxin-Pf6/1-36 AECRWMFGSCKEDSDCCKHLGCRRDAPQYCAWDGTV
U4-theraphotoxin-Pf7/1-37 EVVLDSEQNRDCTKLLGGCKTDAECCPHLGCKVMCSQ
U4-theraphotoxin-Pf8/1-37 DCTKLLGGCKTDAECCPHLGCRKKWPYHCGWDGPSDK
U4-theraphotoxin-Pf9/1-37 ECRQFWGWCSRDSDCCKHLSCKRKWPNICLWDGTFTK
U4-theraphotoxin-Pf10/1-37 EMFSLIAEGVCWCVDKTGRVLTNHQGHNIMLTFEIQS
U4-theraphotoxin-Pf11/1-39 KKECSQLLGSCTKDSDCCSPFSCTPKWPRYCSWHSIFQI
U4-theraphotoxin-Pf12/1-39 GCKTDAECCPHLGCRMGKFEAHWNTTCTTRSAPDSKSPS
U4-theraphotoxin-Pf13/1-34 ECRYWLGGCEKTSDCCEHLSCSPKHGWCVWDWTF
U4-theraphotoxin-Pf14/1-36 DCKQLFGTCKKDEECCEHLGCNKKYGWCGWDGTFGR
U4-theraphotoxin-Pf15/1-36 ECRWYLGACKKDSDCCKHLQCHSYVGWCIWDGTQGQ
U4-theraphotoxin-Pf16/1-36 ECRYWLGGCEKTSDCCEHLSCSPKHGWCVWDWTFRK
U4-theraphotoxin-Pf17/1-37 MRCFSAMFSAEDQLSPQEKGMSDCISLYDSFFYHNLL
U4-theraphotoxin-Pf18/1-37 LCKSDDDCCPHLMCYKYGWCGWDGSVGGSASLLLPNC
U4-theraphotoxin-Pf19/1-38 FSAEDQLSPQERECRYWLGGCEKTSDCCEHLSCSPSVC
U4-theraphotoxin-Pf20/1-41 ECRYFWGECNDKDLVCCDYLVCKYKWPLSYNICVWNRTFPG
U4-theraphotoxin-Pf21/1-31 NCAKEGELCGWGSRCCHDLYCPAAVVAYCEP
U4-theraphotoxin-Pf22/1-34 CYASEVEELNLQDEDCNIYSDSCAGSVCRPFSWM
U4-theraphotoxin-Pf23/1-36 QEYVPETENCAKEGELCGWGSRCCHDLYCPAALSDM
U4-theraphotoxin-Pf24/1-37 ESERASACAKEGEVCGWGKRCCDLDNYYCPAGIVPFC
U4-theraphotoxin-Pf25/1-36 ECRYFWGQCGGQEGNCCAHLVCRRKWPNICIWDLTV
U4-theraphotoxin-Pf26/1-37 ECRYFWGQCGGQEGNCYFVKLNFSACNCCKSQTLPLH
U4-theraphotoxin-Pf27/1-33 WYLGACKKDSDCCKHLQCHSYWEWCIWDGKVTF
U4-theraphotoxin-Pf28/1-33 MCHNKEFMGLCKSDDDCCPHLMCYKYGWCGWDG
U4-theraphotoxin-Pf29/1-34 CSRYFLGGCTEHSDCCEHLSCKMGLNYCAWDGTF
U4-theraphotoxin-Pf30/1-34 GGCTEHSDCCEHLSCKMESQYKLFTMHCSICPEN
U4-theraphotoxin-Pf31/1-35 ECRWYLGACKKDSDCCKHLQCHSYWEWCIWDGTIS
U4-theraphotoxin-Pf32/1-35 AGCRYFLGGCTEHSDCCEHLSCKMGLNYCAWDGTF
U4-theraphotoxin-Pf33/1-39 IFECSLSCDIKKEGKACKGKGEKKCGGGWRCKMNFCLKF
U4-theraphotoxin-Pf34/1-39 IFGCTLSCDIKKEGKACKGKGEKKCGGGWRCKFNFCIKF
U4-theraphotoxin-Pf35/1-42 CIGEGVPCDENDPRCCSKLECLKPKGYGWWYKSYYCYKKKS
P61790_Omega-sparatoxin-Hv1b_Heteropoda_venatoria/1-36 ADCGWLFHSCESNADCCENWACATTGRFRYLCKYQI
Conservation
000000026332329+125441312200000
Quality
Consensus
DLGDA+FSAEDQ+SEQEEECR++LGGCKKDSDCCKHLGCKK+G++CCWWDTFV+KF++SS++PS
