# Supplementary material for: Peptidomic and transcriptomic profiling of four distinct spider venoms
Source: PLoS One. 2017 Mar 17;12(3):e0172966. doi: 10.1371/journal.pone.0172966 (PMC5357004; doi:10.1371/journal.pone.0172966)
Supplement: S7 Fig — The last reference sequence is the Kappa-ctenitoxin-Pn1a from Phoneutria nigriventer (Ctenidae) (Uniprot, accession number O76200). (PDF) [file pone.0172966.s014.pdf]

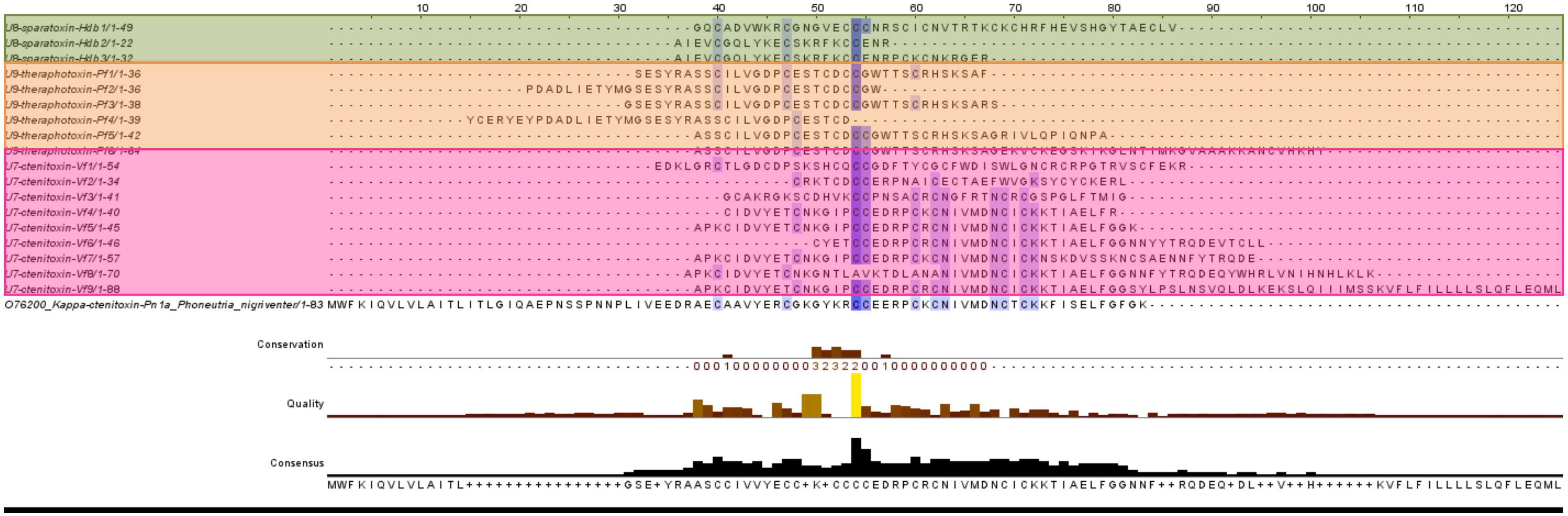
U8-sparatoxin-Hdb1/1-49
U8-sparatoxin-Hdb2/1-22
U8-sparatoxin-Hdb3/1-32
U9-theraphotoxin-Pf1/1-36
U9-theraphotoxin-Pf2/1-36
U9-theraphotoxin-Pf3/1-38
U9-theraphotoxin-Pf4/1-39
U9-theraphotoxin-Pf5/1-42
U7-ctenitoxin-Vf1/1-54
U7-ctenitoxin-Vf2/1-34
U7-ctenitoxin-Vf3/1-41
U7-ctenitoxin-Vf4/1-40
U7-ctenitoxin-Vf5/1-45
U7-ctenitoxin-Vf6/1-46
U7-ctenitoxin-Vf7/1-57
U7-ctenitoxin-Vf8/1-70
U7-ctenitoxin-Vf9/1-88
O76200_Kappa-ctenitoxin-Pn1a_Phoneutria_nigriventer/1-83
Conservation
Quality
Consensus
